# Medulloblastoma Exosome Proteomics Yield Functional Roles for Extracellular Vesicles

**DOI:** 10.1371/journal.pone.0042064

**Published:** 2012-07-27

**Authors:** Laura M. Epple, Steve G. Griffiths, Anjelika M. Dechkovskaia, Nathaniel L. Dusto, Jason White, Rodney J. Ouellette, Thomas J. Anchordoquy, Lynne T. Bemis, Michael W. Graner

**Affiliations:** 1 Department of Neurosurgery, Anschutz Medical Center, University of Colorado Denver, Aurora, Colorado, United States of America; 2 Cell and Molecular Biology Program, Cancer Biology, College of Veterinary Medicine and Biomedical Sciences, Colorado State University, Fort Collins, Colorado, United States of America; 3 Atlantic Cancer Research Institute, Moncton, New Brunswick, Canada; 4 Department of Surgery, Duke University Medical Center, Durham, North Carolina, United States of America; 5 School of Pharmacy, Anschutz Medical Center, University of Colorado Denver, Aurora, Colorado, United States of America; 6 Department of Medical Oncology, Anschutz Medical Center, University of Colorado Denver, Aurora, Colorado, United States of America; University of Navarra, Spain

## Abstract

Medulloblastomas are the most prevalent malignant pediatric brain tumors. Survival for these patients has remained largely the same for approximately 20 years, and our therapies for these cancers cause significant health, cognitive, behavioral and developmental sequelae for those who survive the tumor and their treatments. We obviously need a better understanding of the biology of these tumors, particularly with regard to their migratory/invasive behaviors, their proliferative propensity, and their abilities to deflect immune responses. Exosomes, virus-sized membrane vesicles released extracellularly from cells after formation in, and transit thru, the endosomal pathway, may play roles in medulloblastoma pathogenesis but are as yet unstudied in this disease. Here we characterized exosomes from a medulloblastoma cell line with biochemical and proteomic analyses, and included characterization of patient serum exosomes. Further scrutiny of the proteomic data suggested functional properties of the exosomes that are relevant to medulloblastoma tumor biology, including their roles as proliferation stimulants, their activities as attractants for tumor cell migration, and their immune modulatory impacts on lymphocytes. Aspects of this held true for exosomes from other medulloblastoma cell lines as well. Additionally, pathway analyses suggested a possible role for the transcription factor hepatocyte nuclear factor 4 alpha (HNF4A); however, inhibition of the protein’s activity actually increased D283MED cell proliferation/clonogenecity, suggesting that HNF4A may act as a tumor suppressor in this cell line. Our work demonstrates that relevant functional properties of exosomes may be derived from appropriate proteomic analyses, which translate into mechanisms of tumor pathophysiology harbored in these extracellular vesicles.

## Introduction

Pediatric tumors of the central nervous system (CNS) are the leading cause of cancer-related mortality in children [1]. Among those tumors, medulloblastomas are the most prevalent malignant pediatric brain tumors [2]. With stratification into different risks groups taken into account, overall survival for patients with medulloblastoma has remained at 70%–80% for approximately 20 years [3], [4]. This survival rate comes at significant cognitive, behavioral, and general physical cost to the surviving patients, as the developmental sequelae are often devastating [5], [6]. Clearly, we need a better understanding of the biology of these tumors, and while great efforts have gone into the molecular genetics of medulloblastomas [7], one area that remains completely unstudied is that of medulloblastoma exosomes.

Exosomes are exocytically-released 30–100 nm diameter membrane-enclosed vesicles derived from the endosomal system during multivesicular body (MVB) formation [8]. MVBs and their contents are often degraded by lysosomes; however, some MVBs fuse with the plasma membrane, releasing their interior vesicular contents into the extracellular space, and these vesicles are then called exosomes. Exosome release and trafficking has implications for extracellular (*in trans*) signaling [9], [10], deportation of toxic substances including chemotherapeutic agents [11], viral passage [12], and horizontal mRNA/microRNA transfer [13]. Exosomes may increase tumor proliferation [14], play roles in the generation of pre-metastatic niches [15], and have divergent effects on the immune response [16], [17]. For medulloblastomas in particular, their migratory behavior is a poor prognostic indicator [18] with dissemination leading to meningeal spread and tumor cell proliferation [19]. In addition, the patients may also display reduced immune responses [20], [21]. These are all factors that may be related to tumor-derived exosomes, including those found in serum; thus, any improvement in our understanding of the complicated activities of these extracellular vesicles may lead to clues about targeting the vital biology of exosomes.

In this report we show that medulloblastoma cell lines produce exosomes with physical and biochemical characteristics similar to those of other brain tumor exosomes [17]. In novel studies for medulloblastoma exosomes we reveal proteomic results that again are consistent with our previous work [17] but we extend those findings to suggest areas of particular functional relevance. We show in those functional studies that these exosomes can drive tumor cell proliferation, enhance migration, and modulate T cell responses in vitro. We also show that a transcription factor associated with hepatic development and tumor biology, HNF4A, is a prominent hub in the proteomic analyses. However, a drug targeting that protein failed to impact tumor cell survival, and may have demonstrated a role for HNF4A as a tumor suppressor. We also show that tumor exosomes are components of sera from patients with medulloblastomas, and that these vesicles possess canonical and unique proteins. The work presented here is partly in response to a perception in the area of brain tumor proteomics that despite extensive efforts, little functional significance has surfaced from the compilation of numerous studies [22]. We hope this demonstrates that appropriate and creative analyses of data can indeed lead to functional implications with relevance for medulloblastoma and other tumor biologies.

## Results

### Biophysical Characteristics of Medulloblastoma Cell Line Exosomes

**Figure 1:**
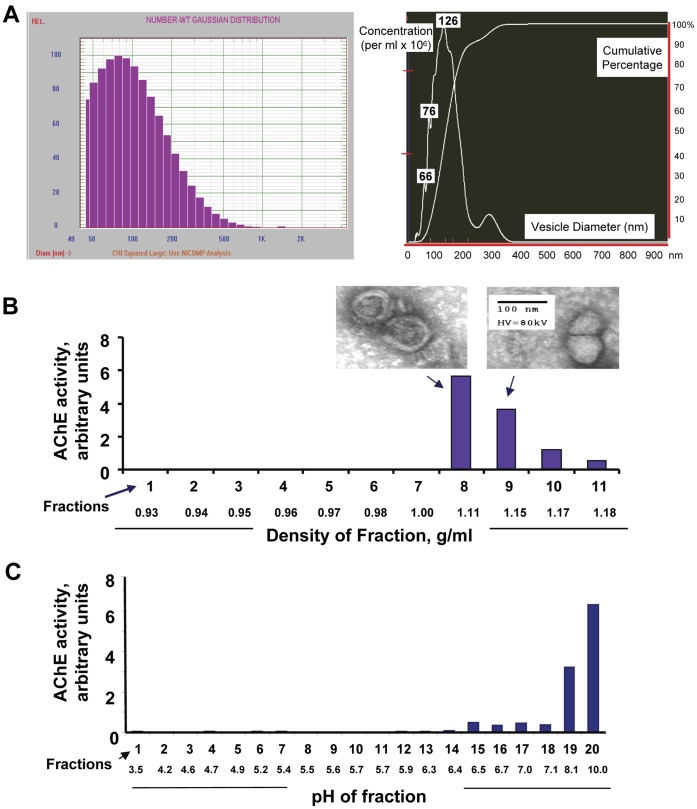
Biophysical characterization of D283MED exosomes. Exosomes were collected from the spent medium of the medulloblastoma cell line D283MED by filtration and differential centrifugation. Vesicles were analyzed by dynamic light scattering (DLS) (**A, left**) and nanoparticle tracking with a NanoSight device (**A, right**) for vesicle diameters and concentration. Sizes at identified peaks are listed. Accounting for dilution, exosome concentration in this case was 2.84×10^8^ particles/ml. In (**B**), D283MED exosomes were subjected to density-gradient centrifugation thru a 0%–60% Opti-Prep step gradient; fractions were collected and densities determined. Fractions containing exosomes were identified by acetylcholinesterase (AChE) activity and electron microscopy (micrographs in inset above peak fractions 8 and 9; bar = 100 nm). Exosomes were also fractionated by Rotofor free-solution isoelectric focusing (**C**) as described in the Materials and Methods. Fractions were harvested and the pH of each was determined. Exosome-containing fractions were again identified by AChE activity.

We harvested exosomes from the spent serum-free media (cells were grown in “knockout” medium supplemented with serum replacement) from the human medulloblastoma cell line D283MED (and for functional studies, from DAOY and UW228). Exosomes were collected by filtration through a 0.22 µm filter, centrifugal concentration, and pelleting at 100,000 x g. We determined the range of vesicle diameters using dynamic light scattering with number-weight Gaussian distribution (Figure 1 A, left). This revealed a mean particle size of ∼100 nm, including bins with many smaller vesicles and some larger, indicative of “microvesicles” [23] or probable clumping of the sample [24] (see also the electron micrographs in Figure 1 B). Particle tracking using Brownian motion to determine vesicle sizes was also done using a NanoSight device [23], [25] (Figure 1 A, right), which revealed a relatively uniform size distribution of peaks at 66, 76 and 126 nm and a dilution-adjusted concentration of 2.8×10^8^ particles/ml. As we and others have done before [17], [26], [27], we utilized density gradient centrifugation to isolate exosomes, and demonstrated their presence by acetylcholinesterase (AChE) activity and by transmission electron microscopy (Figure 1 B, and insets). The fraction densities and associated AChE activities were consistent with exosomes isolated from other cell types including murine brain tumor cells [17], [26], [27]. We also characterized the isoelectric point of the medulloblastoma exosomes, and found them to be nearly identical to those our previously characterized murine brain tumor exosomes [17] with very basic isoelectric points (Figure 1 C). This property may be related to tumor microvesicle zeta potential, which was found to be relatively high and negative for tumor vesicles [24].

### D283MED Exosome Proteomics Reveal Tumor- and Disease-related Networks

**Figure 2:**
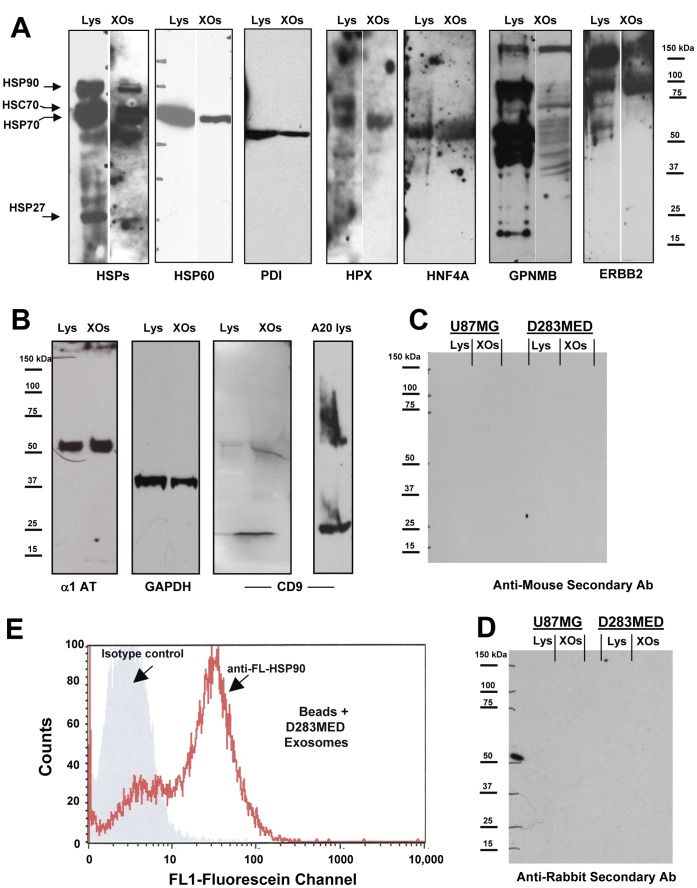
Western blot and FACS analyses of D283MED exosomes. Exosomes harvested from the spent medium of D283MED cells, and the cells themselves, were lysed and proteins separated on SDS-PAGE gels followed by electrotransfer for Western blotting and probing with the antibodies listed. (**A**) shows blots probed for chaperone proteins, a potential tumor transcription factor, and known brain tumor antigens. Results of probes for heat shock proteins (HSPs) 90, 70, 27, and 60 (and heat shock cognate 70–HSC70), as well as protein disulfide isomerase (PDI) and hemopexin (HPX), hepatocyte nuclear factor alpha (HNF4A), and tumor antigens glycoprotein non-metastatic B (GPNMB) and Her2/Neu (Erb B2 : ERBB2) are shown (**B**) shows blots probed for proteins typically found in exosomes such as alpha-1 antitrypsin (α-1AT), glyceraldehyde 3-phosphate dehydrogenase (GAPDH), and the exosome marker CD9. A20 (murine leukemia/lymphoma cell line) lysate is a positive control for CD9. (**C, D**) Control blots of exosomes and lysates listed were probed with anti-mouse and anti-rabbit secondary antibodies only (respectively). Molecular weight markers are indicated at the sides of the blots. Exosome surface HSP90 was identified by fluorescence activated cell sorting (FACS) analysis of exosomes bound to latex beads and treated as if they were cells in FACS (**E**). Gray fill indicates fluorescence of exosome-coated beads probed with a fluorescently-labeled isotype control antibody, and the red line shows fluorescence intensity of the exosome/bead complex with the fluorescently-labeled anti-HSP90 antibody.

**Figure 3:**
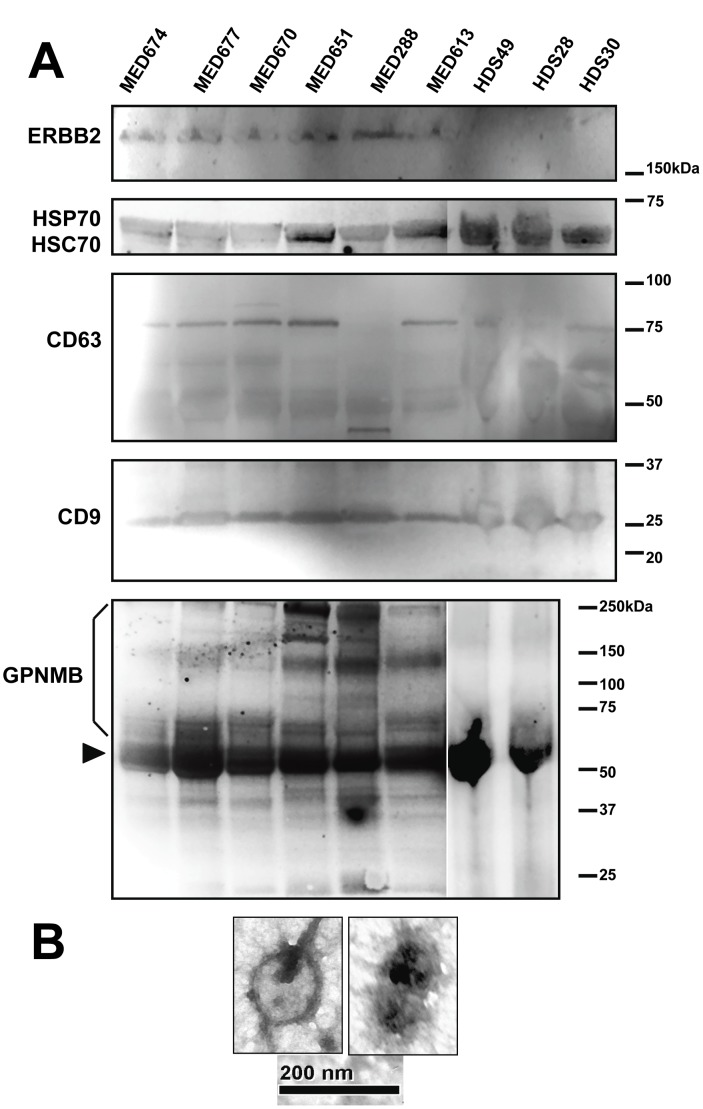
Western blots and TEM of serum exosomes from medulloblastoma patients and healthy donors. Exosomes from sera from patients with varying medulloblastoma subtypes (denoted as MEDxxx) and 3 healthy donors (HDxx) were precipitated using ExoQuick solution. (**A**) Vesicles were lysed according to the manufacturer’s protocol and proteins separated on SDS-PAGE gels followed by electrotransfer for Western blotting and probing with the antibodies listed. Note that ERBB2 and heterogeneous (glycol)forms of GPNMB seem to show specificity for exosomes from patients only. Arrowhead in the GPNMB blot shows either the predicted GPNMB core protein or else a non-specific band also found in the healthy donor lanes. (**B**) Transmission Electron Microscopy (TEM) micrographs of medulloblastoma serum exosomes precipitated with ExoQuick.

We performed gel-based separations of exosome proteins with in situ protease digestion of gel slices to obtain peptides for mass spectrometry mapping and de novo sequencing for protein identity. We identified 148 proteins (of those that were matched in two separate runs with MOWSE scores ≥35 (via Mascot) or ≥95 for MS/MS peptide ion fragment matches; other proteins were included on the basis of their presence via Western blotting (Figure 2, and [17]) as listed in Table S1). Of those, 145 were trackable in Ingenuity Pathway Analysis software and are shown in Table S1 (one protein was redundant–HSP70A1A/B, equivalent to HSP70A1B–and two Ig kappa and lambda chains lacked appropriate gene symbols but were grouped as immunoglobulins). The Table also indicates whether the proteins we identified have been previously entered into the ExoCarta database, arguably the most comprehensive protein/mRNA listing of published information on the proteomics and RNA content of exosomes (http://www.exocarta.org/). Comparing our data to those in ExoCarta, we found that 51 (over 35%) of our MS- and immuno-identified proteins were not yet listed and may be novel entries into the database. However, some of the proteins have close homologues (such as kinesin family members or the hnRNPs) that are listed in the database, so it is likely that the classes of proteins overlap. Western blotting (Figure 2) confirms the presence of chaperones (eg, the canonical HSP/HSC70, HSPs 90, 60 and 27, and the endoplasmic reticulum resident and cell surface chaperone protein disulfide isomerase), including the hemolysis cytoprotectant hemopexin. In addition, we found the tumor transcription factor HNF4A, and signaling molecules/tumor antigens GPNMB and ERBB2 (Her2/neu) (Figure 2 A), and molecules commonly associated with exosomes (Figure 2 B), including the tetraspanin CD9. We had previously demonstrated that HSPs 25/27 and 70 were on the surfaces of brain tumor-derived exosomes [17], and here we show that HSP90 is displayed on D283MED exosome surfaces as determined by flow cytometry (Figure 2 E). To our knowledge, this is the first such indication of exosome surface HSP90. Control blots using anti-mouse and anti-rabbit secondary antibodies, respectively (Figure 2 C,D) reflect primary antibody specificity. We extended our Western blotting analyses to serum exosomes from patients with medulloblastomas compared to those from healthy donor sera (Figure 3 A), showing reactivity with patient serum exosomes against ERBB2, and GPNMB (both brain tumor antigens), but general reactivity against canonical exosome markers HSP/HSC70, CD63, and CD9. GPNMB is a complex glycoprotein with multiple glycoforms in brain tumor cells as seen in previous Western blotting [28] including those from murine brain tumor exosomes [17]. For the GPNMB probe note the presence of a strong signal in the precipitated exosomes from either patient sera or healthy donor sera (arrowhead). This may be non-specific binding, but it is in the molecular weight range (∼60 kDa) of the predicted GPNMB core protein. However, the heterogeneity is evident in the exosomes from patient sera, and the general pattern differs clearly between the two exosome sources. We verified the presence of exosome-like vesicle in the preparations by transmission electron microscopy (Figure 3 B).

**Figure 4:**
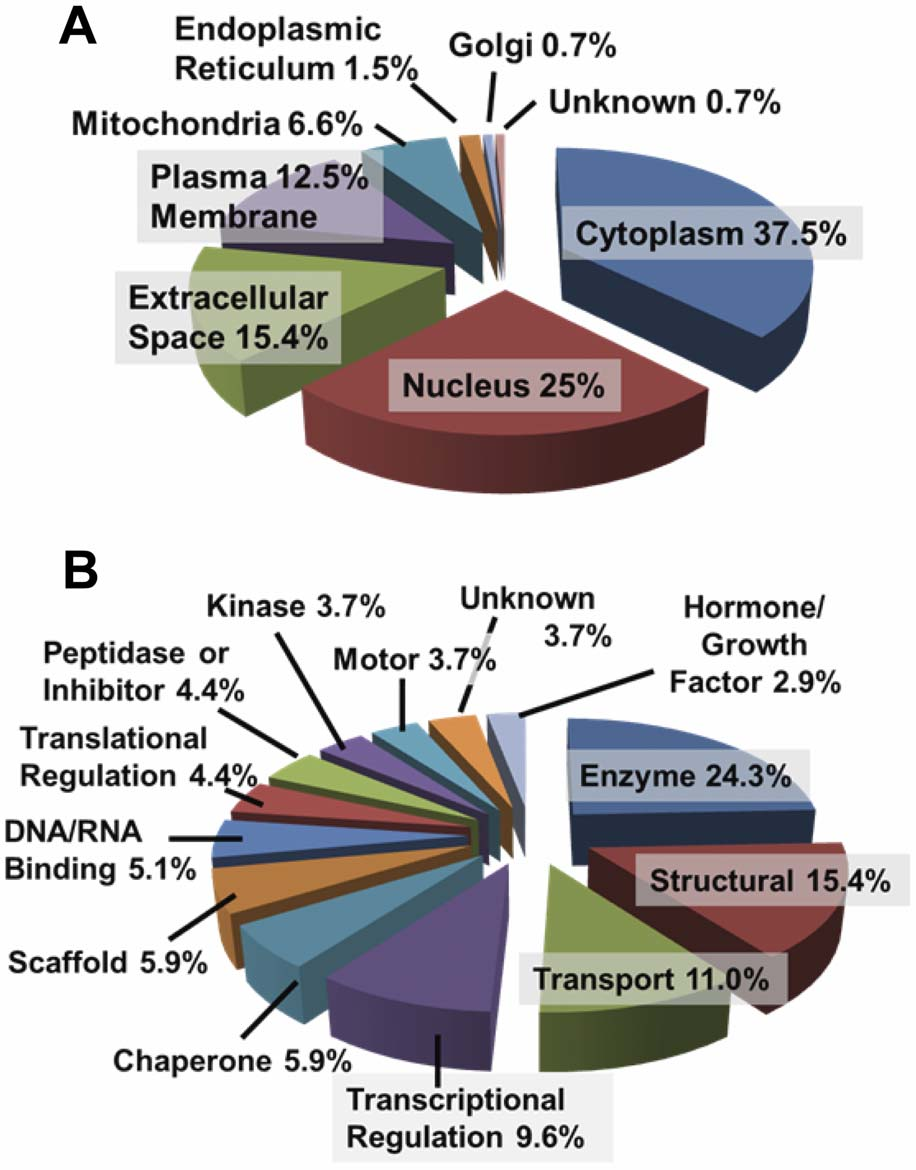
Categorization of D283MED exosomal proteins by subcellular localization and by function. The proteins listed in Table S1 were categorized as percentages of the total number of proteins identified using Ingenuity Pathway Analysis descriptions and literature searches. Proteins are classified by subcellular (or extracellular) localization (**A**) or by function (**B**).

We categorized the original subcellular/extracellular localizations of the exosomally-derived proteins identified from Table S1 using databases and literature searches. The proteins identified are associated with most of the major cellular organelles and subcellular/extracellular localizations (Figure 4 A). These distributions indicate high proportions of cytosolic- and nuclear-derived proteins, with relatively lower percentages of plasma membrane and extracellularly-localized proteins. There were also a few endoplasmic reticulum residents, and one protein each of Golgi or of unknown subcellular localization. While we saw a reduction in percentages of cytoplasmic proteins compared to our previous work [17], here we found more than twice the percentage of nuclear proteins (>26%), and we also included mitochondrial-derived proteins as a category (6.9%), which may have reduced the overall proportion of cytoplasmic entities. The percentage of unknown proteins was reduced compared to our previous report, probably due to the improved correlations between genes identified and actual proteins being studied. As a caveat, it must be stated that numerous proteins we identified have multiple subcellular localizations, particularly in tumors; for example, the chaperones of the HSP70 (including GRP78) and HSP90 families, as well as HSP27 and protein disulfide isomerase, may translocate to the nucleus, the cytoplasm, and even the cell surface [29], [30], [31]. The proteins run the gamut of activities and functions, including cytoskeletal and structural components, nucleic acid-binding proteins, transcriptional and translational regulators, transporters, chaperones, kinases and signaling components, and a wide variety of enzymes (Figure 4 B). Functionally, the largest single category of proteins could be grouped as enzymes, with nearly the same percentage as seen previously. Transcriptional regulators, transport proteins, and structural proteins combine for over one-third of the remaining functions, with chaperones, nucleic acid binding proteins, scaffold proteins and proteins of unknown function holding similar percentages. The lowest represented functions were proteases/inhibitors, translational regulators, motor proteins, kinases, and hormones. A similar caveat applies in that many of these proteins are multifunctional and may play multiple roles, particularly in complexes, thus making definitive categorization difficult.

Using Integrated Pathway Analysis software, the identified proteins were grouped into networks of associated functions, canonical pathways, and disease and toxicology relationships (Figures S1, S2, S3). The top 5 networks/associated functions were “Cell Morphology, Post-Translational Modification, Protein Folding” (score = 48); “Genetic Disorder, Hematological Disease, Renal and Urological Disease” (score = 39); “Carbohydrate Metabolism, Energy Production, Nucleic Acid Metabolism” (score = 37); “Neurological Disease, Genetic Disorder, Hematological Disease” (score = 34); “Carbohydrate Metabolism, Gastrointestinal Disease, Genetic Disorder” (score = 27). The scores (-log [p values]) reflect the probabilities of such associations occurring by chance, with the threshold value for significance set at 1.25; as evident, the scores are highly significant. Figure S1 lists the 72 significant biological functions within the “Disease and Disorders” category; Figure S2 shows the 28 significant-scoring Canonical Pathways, and Figure S3 shows the significant-scoring Toxicology and Toxicology Function Lists.

**Figure 5:**
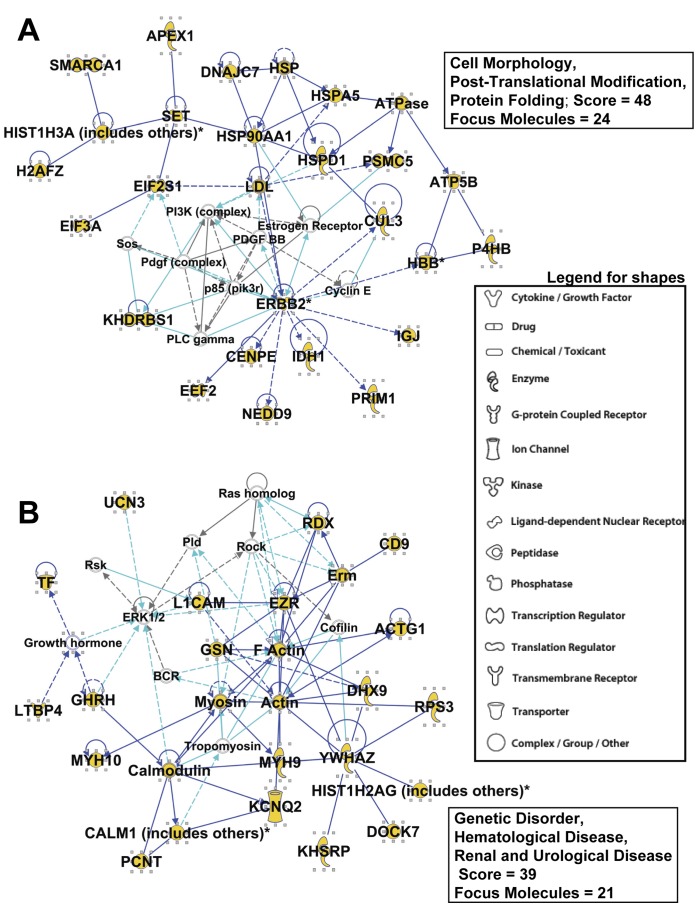
Interactomes of the Top Networks/Associated Functions from IPA “Core Analysis”. Proteins clustered within the Top Networks/Associated Functions as derived from IPA algorithms are shown as members of “interactomes”. Proteins identified during this work are labeled in larger bold font, with the protein symbol in gold fill. Direct connections between/among proteins are shown in solid lines; indirect interactions are shown as dashed lines (also called “edges”). Connections between proteins identified in this proteomic screen are shown in dark blue; interactions between proteins we identified and proteins not identified in our proteomics are shown in turquoise. Protein shapes are indicative of function and that legend is shown in **Figure 5** **A,B**. Each network’s score (Fisher’s exact test, -log [p values] shown; all networks were highly significant) and number of focus molecules (those which are “seeds” for generation of focal points within the network) are shown. The top 2 network terminologies are: (**A**) “Cell Morphology, Post-Translational Modification, Protein Folding”; (**B**) “Genetic Disorder, Hematological Disease, Renal and Urological Disease”;

Figure 5 shows the “interactomes” of the top 2 (out of 16 significant-scoring) local connecting networks and functional associations within those networks. Proteins identified in the proteomics (and generic classifications of those molecules) are shown in larger font with yellow highlights; direct connections (ie, known or documented interactions) between identified proteins are indicated by solid blue lines, and indirect connections (either suspected or known via intermediaries) are shown with broken lines. Light blue/turquoise lines show identified molecular interactions with proteins in the network, but we did not specifically identify those other proteins. The network score (related to significance as mentioned above) and the numbers of focus molecules are shown, along with the inset legend for the shapes related to molecular functions. Figure 5 A (“Cell Morphology, Post-Translational Modification, Protein Folding”) demonstrates the extraordinary connectivity of the chaperone protein system with that of the PI3K, PDGF, and ERBB2 signaling complexes, and translation systems, possibly manifesting in cell proliferation. ERBB2 is a known client protein of the chaperone HSP90 [32] and as such has been one of the oncogenic targets of HSP90 inhibitors [33]. As an extracellular HSP (eg, see Figure 2 E), it interacts with ERBB2 to promote tumor cell invasion [34]. A similar set of proteins has been identified as differentially expressed in ERBB2+ breast cancer [35]. Figure 5 B (“Genetic Disorder, Hematological Disease, Renal and Urological Disease”) shows interesting linkages from extracellular and cell surface localizations to intracellular signaling and cytoskeletal/motor units, suggesting roles in cell movement and migration. Thus, these interactomes have clear relevance to the cancer state, and often overlap with elements of intravesicular trafficking and extracellular activites. As such, the pooled protein components may suggest functional features of exosomes as they play roles in medulloblastoma biology.

### Medulloblastoma Exosome Proteomics Suggest Exosome Functions

**Figure 6:**
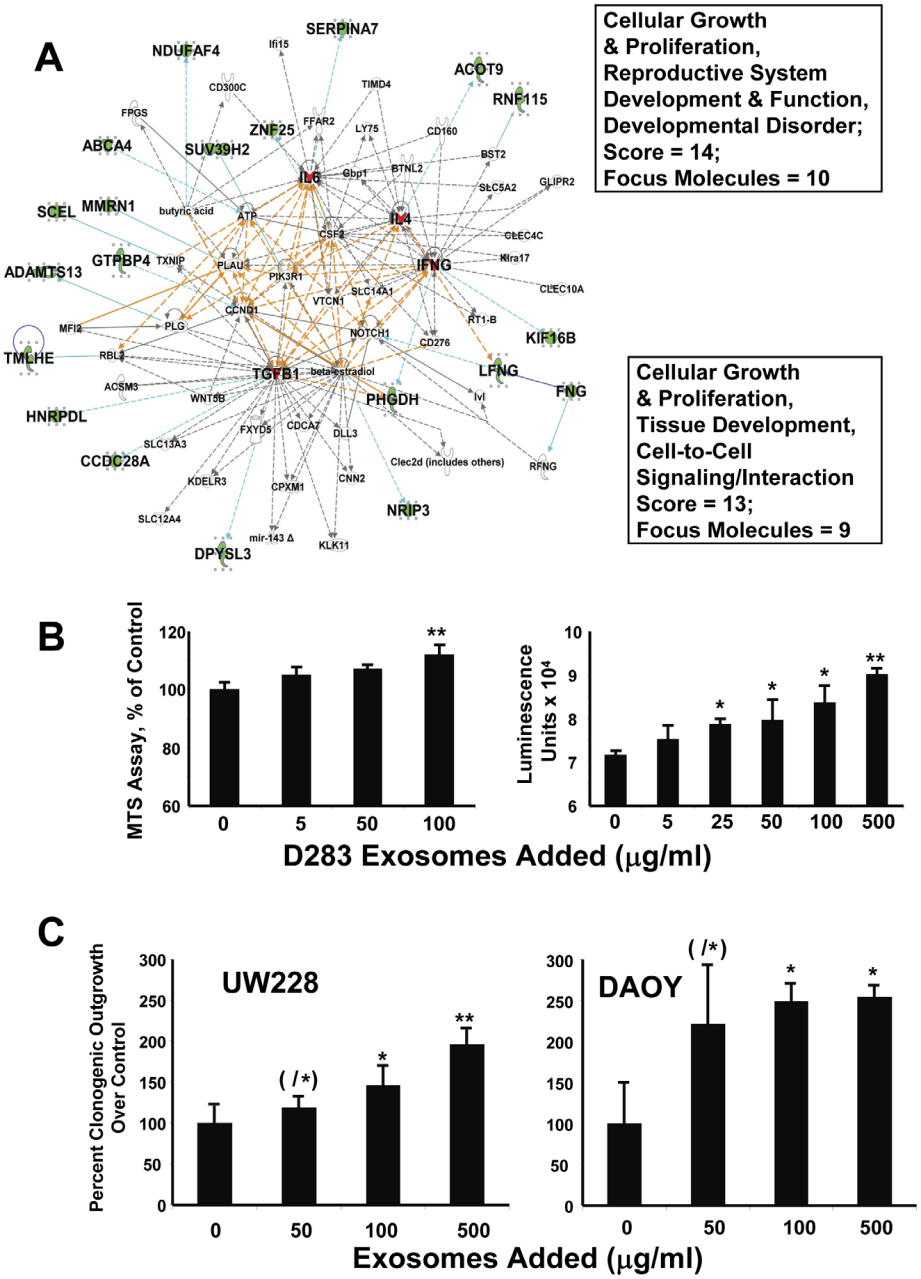
Exogenous exosomes promote tumor cell proliferation. IPA Networks 7 and 8 in combination (**A**) suggested that exosomes may provide cell growth stimulation (boxed terms and scores for each work are show at right). Networks are represented as described for **Figure 5**, with overlapping connections shown in orange. Proteins identified in our studies are shown in green fill here to stand out against the orange lines. Exosome-driven increases in proliferation (**B**) were measured by MTS assay (**left**) and an ATP assay (**right**), where increasing quantities of exosomes were incubated with D283MED cells in tissue culture resulting in dose-dependent increases in proliferation at 24 (**left**) and 48 hrs (**right**) (see also **Figure 9B** for a clonogenic analysis of increased proliferation following exosome stimulation). (**C**) shows quantified clonogenic outgrowths of UW228 and DAOY cells exposed to cognate exosomes. Differences between groups were statistically evaluated by ANOVA; significant differences (p<0.05) between groups are indicated by different “star cluster” numbers (eg, *, **, ***). Control cell proliferation (no exosomes) was set at 100%. For the UW228 experiment, (/*) means that the 50 µg/ml value differed significantly from the 500 µg/ml, but not control (0 µg/ml) or 100 µg/ml value. For the DAOY experiment, (/*) means that means that the 50 µg/ml value differed significantly only from the control (0 µg/ml).

The proteins within the various functional networks imply specific activities that may be inherent in D283MED exosomes. Interactomes/networks 7 and 8 both suggested impacts on cell growth and proliferation (combining interactomes shown in Figure 6 A–connections linking the interactomes are indicated as orange lines). Thus, we asked if adding exogenous medulloblastoma exosomes to medulloblastoma cells in culture would promote cell proliferation. This appears to be true, as exosomes from the tumor cells enhance proliferation of the cells of origin in culture in a dose-dependent manner (Figure 6 B, C). As measured by both MTS assay and ATP assay (Figure 6 B, left and right graphs), significant increases in metabolic outputs occurred after 48 hrs incubation. As D283 cells are perhaps the slowest growing of the published medulloblastoma lines [36], and exosomes increase recipient cell metabolic activity (Epple et al, unpublished), it was possible that the effects we saw were those of increased metabolic rate and not necessarily enhanced proliferation. Thus, we verified by clonogenicity assays that exosomes did indeed increase cell proliferation not just for D283 cells but also for cell lines UW228 and DAOY (Figure 6 C; see a later figure for results with D283 cells).

**Figure 7:**
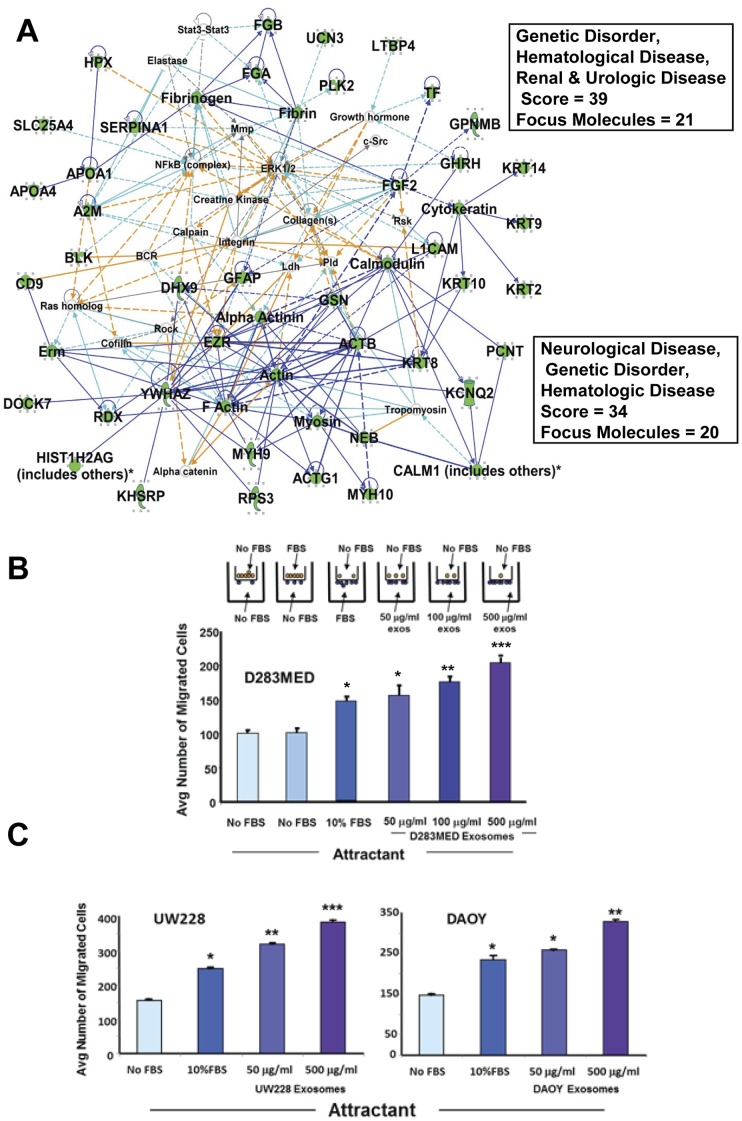
Exogenous exosomes are attractants for tumor cell migration. IPA Networks 2 and 4 in combination (**A**) suggested that exosomes may provide impetus for tumor cell migration (boxed terms and scores for each work are show at right). Networks are represented as described in **Figures 5** and **6**. (**B**) depicts the set-up for a Boyden chamber type of migration assay (**top**) and results (**bottom**). D283MED cells were placed in the upper chambers and attractants (10% fetal bovine serum [FBS] as positive control, media only [no FBS] as a negative control, or increasing concentrations of D283MED exosomes) were added to the lower chambers. Cells were separated from the lower chamber by a polycarbonate (8 µm pore size) filter. After 48 hrs, cells that migrated thru the insert were stained and counted in 3 microscope fields (average per field +/− standard deviation shown). The same assays were performed using UW228 and DAOY medulloblastoma cell lines and exosomes (**C**), **left** and **right**, respectively. Differences between groups were statistically evaluated by ANOVA; significant differences (p<0.05) between groups are indicated by different “star cluster” numbers (eg, *, **, ***).

The protein cohort in Figure 5 B, particularly when combined with Network 4 (Figure 7 A) strongly suggests cell migration given the number of structural/cytoskeletal components and the roles of L1-NCAM and EZR in cell migration and tumor metastases [37], [38]. We tested this assertion by using medulloblastoma cell line exosomes as attractants in a minimal Boyden chamber assay; medulloblastoma cells were placed atop a plastic membrane (8 µm pore size) in the upper chamber while serum-free medium (negative control), 10% fetal bovine serum (positive control), or increasing concentrations of exosomes were placed in the lower chamber to serve as attractants. As seen in Figure 7 B, exosomes promote tumor cell migration in a concentration-dependent fashion which is minimally as good as, or better than, FBS and at higher exosome concentrations is significantly better. Whether the cells were serum-starved or not is of no consequence in terms of baseline migration. The migration of D283MED cells through what was essentially naked plastic towards exosomes is impressive given the lack of adherence of these cells to commonplace matrix substrates [39]. Adherent medulloblastoma cell lines (when grown in FBS-containing medium) UW228 and DAOY (Figure 7 C) also migrate towards cognate exosomes in a dose-dependent fashion.

**Figure 8:**
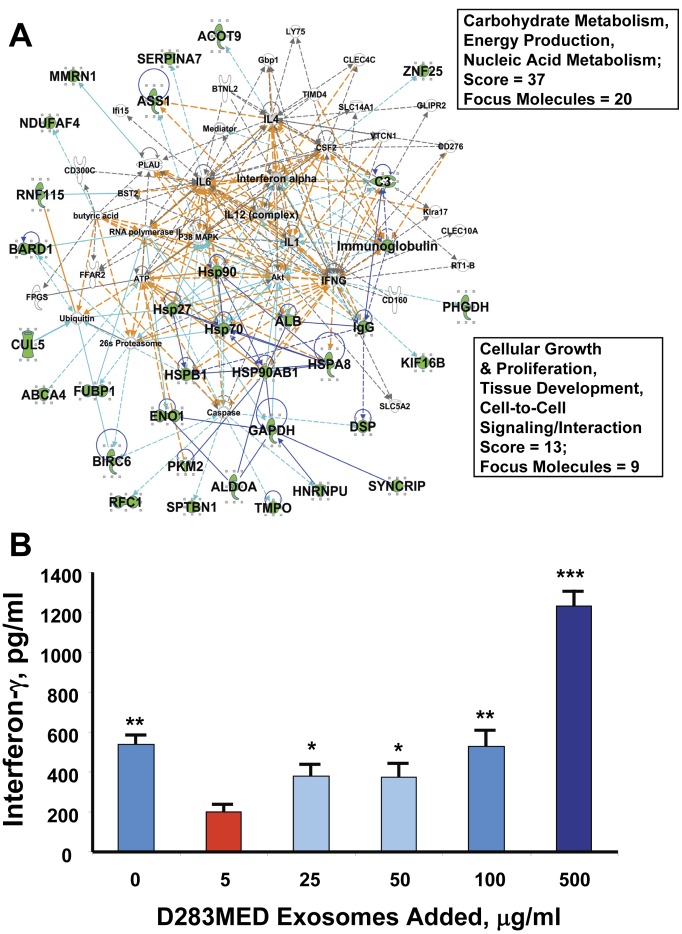
D283MED exosomes affect interferon-gamma output from activated PBMCs. IPA Networks 3 and 8 in combination (**A**) suggested that exosomes may be involved in immune cell cytokine release (boxed terms and scores for each work are show at right). Networks are represented as described in **Figures 5** **,** **6** and **7**. Immune-related cytokines are also noted in larger font and with gray fill. (**B**) shows exosome-induced changes in PHA-activated PBMCs; healthy donor PBMCs were stimulated with PHA (5 µg/ml) for 48 hrs and D283MED exosomes at the concentrations listed were added as well. Interferon-γ release was measured by ELISA. Differences between groups were statistically evaluated by ANOVA; significant differences (p<0.05) between groups are indicated by different “star cluster” numbers (eg, *, **, ***).

The merged interactomes of Networks 3 and 8 focus on a number of immune-related molecules (IL-1, IL-4, IL-6, IL-12, CSF2, IFNA, IFNG). Given the previous history (and controversy) of exosomes in the immunobiology of tumors [16], [40] we asked if D283MED exosomes could influence interferon-γ (IFNG) release from activated human T cells. Healthy donor T cells were activated to secrete IFNG by phytohemagglutinin (PHA) stimulation in the presence of increasing concentrations of tumor exosomes. As seen in Figure 8 B, there is a dichotomy of IFNG secreted from otherwise-activated T cells following stimulation with PHA, whereby quantities of exosomes from 5 µg/ml up to 100 µg/ml lead to significantly decreased amounts of IFNG (or no change), whereas greater quantities (500 µg/ml) of exosomes actually increase IFNG output from the activated T cells. There are relatively few publications where assays were performed with exosome concentrations that were as high as ours (see [41] for an exception), which raises the question of a critical balance between immune stimulation versus suppression.

**Figure 9:**
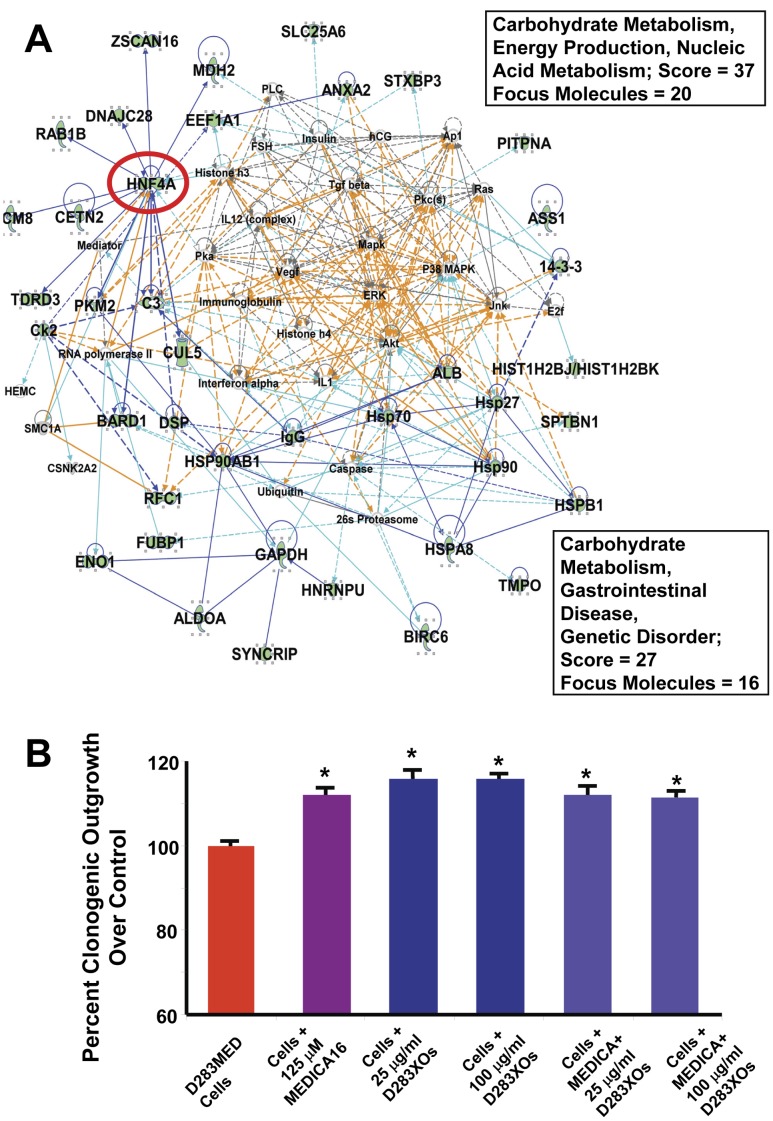
Inhibition of hepatocyte nuclear factor 4α (HNF4A) actually increases D283MED cell growth. IPA Networks 3 and 5 in combination (**A**) reveal that HNF4A (circled in red) sits at a node of interaction with nearly a dozen other proteins in networks tied to cancer cell metabolism (boxed terms and scores for each work are show at right). Networks are represented as described in **Figures 5** **,** **6** **,** **7** **and** **8**. (**B**) D283MED cells were treated with MEDICA16 (125 µM) to inhibit HNF4A; cells were also treated with D283MED exosomes (25 or 100 µg/ml) with or without MEDICA16. Cells were grown in a clonogenic assay for 8 days and were quantified following the various treatments. Differences between groups were statistically evaluated by ANOVA; significant differences (p<0.05) between groups are indicated by different “star cluster” numbers (eg, *, **, ***). Control cell growth (D283MED cells with no drug or exosome treatments) was defined as 100%.

The merged interactomes of Networks 3 and 5 (Figure 9 A) generated a distinct hub around hepatocyte nuclear transcription factor 4 alpha (HNF4A), suggesting its involvement in tumor metabolism and transcriptional regulation. The hypolipidemic and anti-diabetogenic drug MEDICA16 is a known inhibitor of HNF4A activity [42], [43], [44], and given the appearance of a central role for HNF4A, we hypothesized that inhibition of this transcription factor would reduce D283MED proliferation. Thus, we treated the medulloblastoma cells with a range of concentrations of MEDICA16 over varying time courses and saw increased cell proliferation at all but the highest concentrations (1 mM), where carrier effects and drug solubility impacted the results (data not shown). We further carried out soft-agar clonogenicity assays where we added exogenous exosomes as part of the assay (Figure 9 B). While both drug and exosomes modestly but significantly increased clonogenic outgrowth of the treated cells (see also Figure 6 B for corroboration of increased cell proliferation in the presence of added exosomes), combinations of drug and exosomes had no additional impacts on the medulloblastoma cells compared to exosomes or drugs alone. These results suggest that HNF4A may actually be a tumor suppressor in D283MED cells, which is similar to its role as a cell differentiator in hepatocellular carcinoma [45], [46]. Thus, it may be loaded into tumor exosomes as a means of packaging and disposal for removal from the cell.

## Discussion

In this report we demonstrated the presence of medulloblastoma exosomes in the cell lines D283MED, DAOY, and UW228, along with biochemical and TEM evidence of tumor exosomes in sera from patients with medulloblastomas. These exosomes share biochemical and biophysical properties of exosomes from other cell types (including brain tumors [17]), including size, density, canonical heat shock protein content, acetylcholinesterase activity, and extremely basic isoelectric points (Figures 1, 2). Using proteins we identified previously [17], and those identified here by Western blotting and by gel-based proteomics, we have established both the commonality and uniqueness of the medulloblastoma exosome proteome, and with the application of analytical software, have generated logical and functional classifications of the proteins detected (Table S1, Figures 4 and 5, Figures S1, S2, S3). These analyses led to testing of functional roles played by exosomes in proliferation, migration, and immune responses (Figures 6, 7, 8). Further indications from the proteomics suggested an important role for hepatocyte nuclear factor 4 alpha (HNF4A) in medulloblastoma biology, which we attempted to inhibit with a known drug inhibitor, MEDICA 16 (Figure 9). Instead, this resulted in an increase in D283MED cell proliferation, suggesting that the transcription factor may actually be a tumor suppressor in at least this cell line. To our knowledge this is the first report of characterizations and functional delineations of medulloblastoma exosomes, and the first to suggest a role for HNF4A in medulloblastoma biology.

The exosome proteome has received extensive study [47], [48], revealing sets of common proteins, canonical proteins, and those unique to particular exosomes based on their cells of origin. Many of these have been cataloged in the ExoCarta website (http://www.exocarta.org/). With that database as a reference, our results show nearly a 65% overlap in protein identity, with the rest appearing unique. Our classifications (Table S1, Figure 4) showed relatively high percentages of nuclear proteins, but also substantial amounts of proteins involved in transcriptional regulation and with nucleic acid binding properties. The classifications may be somewhat artificial, since as mentioned, proteins like the HSPs appear in multiple subcellular locations. Also, CENPE, a kinetochore protein linking chromatin to microtubules, is considered a nuclear protein, even tho at that point in the cell cycle the nucleus has broken down [49]. The Cullins may associate in complexes with potential nuclear localization, but are not necessarily themselves nuclear localized [50]. Heterogeneous ribonuclear proteins (hnRNPs) are also known to cell surface localize [51]. Thus, rigid classifications may be futile.

The exosome proteome has more often than not been utilized to predict disease biomarkers rather than to predict functional activities of the vesicles [52], [53], [54]. Our own work here with patient serum exosomes implies that GPNMB and/or ERBB2 may be tumor-specific exosome markers (Figure 3). Indeed, one lament in the area of brain tumor proteomics has been the lack of functional association with proteomic data [22]. Herein we showed that exosome biology can derive from analyses of the proteome, demonstrating and validating that tumor-derived exosomes may enhance proliferation of tumor cells, which has been demonstrated previously [55], [56]. This is important because tumor-derived, exosome-like vesicles have been shown to induce apoptosis in a pancreatic cancer cell line [57], so the effects of extracellular vesicles may vary depending on the microvesicles and perhaps the recipient cells.

Exosome-driven migration of endothelial cells has been shown for exosomes of tumor [58] and cardiomyocite progenitor [59] origins, as well as cancer cell exosomes promoting cancer cell migration [60] with an implication for extracellular HSP90 [61], and RAB GTPases [62]. Our proteomic analysis also suggested a role for L1-NCAM (CD171), which has a cleaved form found extracellularly and in exosomes [63], but appears full-length in D283MED exosomes [17] and in another brain tumor cell line [64] where it plays a role in cell motility. Platelet-derived exosomes/microvesicles also have migratory attractant properties towards tumor cells [65], and the vesicles induce MMP activities in recipient cells. Tumor cells themselves secrete active MMPs in exosomes (such as MMP-14, [66]), and other brain tumor exosomes possess active MMPs (N Dusto and L Epple, unpublished). Our results here suggest that exosomes may play an important role in attracting or “leading the way” for medulloblastoma migration; given that the D283MED cell line is not considered very adherent, much less highly migratory in culture [39], the cell migration induced by exosomes in the absence of any sort of matrix is all the more remarkable (Figure 7 B). Medulloblastoma cell lines UW228 and DAOY grow adherently in FBS-containing media and show ample migratory capacity in response to both FBS and exosomes as attractants (Figure 7 C).

Exosomes in the immune response have been heavily studied [16] where there was initial excitement that tumor-derived exosomes might be perfect anti-cancer vaccines, since the vesicles are essentially tiny surrogates of tumors themselves [67], and there is a long history of tumor exosomes as effective vaccines in pre-clinical settings [68] including our own work [17]. There is also a Phase I clinical trial reported from China [69] for colorectal cancer patients that employed autologous exosomes obtained from ascites fluid as a vaccine. However, while the opportunity for artificial (anthropogenic; laboratory manipulation) positive immunomodulation of extracellular vesicles remains eminently possible, the pendulum has unequivocally swung in the other direction in situ: tumor exosomes are now widely regarded as immunosuppressive [70], [71]. We pulsed medulloblastoma exosomes onto activated healthy donor PBMCs and found a dichotomy in the response of the PHA-activated T cells–relatively low doses of tumor exosomes (5–50 µg/ml) led to reduction in IFNG release; 100 µg/ml was statistically neutral, and 500 µg/ml D283 exosomes led to increased IFNG release from the T cells. Since relatively few studies other than Ren et al [41] used exosome suspensions at relatively high concentrations in similar assays, it is possible that other studies essentially “stopped short” of these results. There is a report of exosomes from bronchoalveolar lavage fluid of patients with sarcoidosis inducing pro-inflammatory cytokine release (IFNG and IL-13) from PBMCs, but it is unclear as to what concentrations of exosomes were used [72]. This does beg the question as to what happens in the tumor microenvironment, where we know very little about the concentrations of exosomes, but presumably the concentrations are locally high. However, other studies have demonstrated even higher serum concentrations of tumor exosomes than we used in the assays [73], [74]; in blood, one may envision that antigen-activated T cells are more likely to encounter antigen-bearing exosomes than perhaps in the tumor microenvironment, given the quantities of tumor exosomes in serum. We propose that tumor exosomes may actually be decoys under these circumstances, although the fate of the T cells following tumor exosome encounter in vivo remains unknown.

The transcription factor hepatocyte nuclear factor 4 alpha (HNF4A) is a member of a nuclear receptor superfamily that plays critical roles in liver development [75] and its expression may be deregulated in tumors [76]. In the liver it regulates a variety of genes in metabolic pathways, and as such is considered a potential drug target [77] in diabetes [78] and metabolic syndrome [79]. One known compound that suppresses HNF4A activity is 3, 3, 14, 14-tetramethylhexadecanedioic acid, a β,β’-dimethyl hexadecanedioic acid called MEDICA 16 [44], [80]. Given its use as an anti-diabtetogenic and hypolipidemic agent [42], [43], we treated D283MED cells with MEDICA 16 based on the position of HNF4A at a hub of direct and indirect relationships with critical molecules in transcription/translation and cancer biology (Figure 9 A). Surprisingly, drug treatment led to an increase in cell proliferation comparable to that of exosome treatment (Figure 9 B), suggesting that HNF4A may play a role as a tumor suppressor in this medulloblastoma cell line. It is possible that HNF4A may serve a purpose for tumors in terms of metabolic responses, but eventually becomes exosome cargo for disposal.

We have thus shown that logical analyses of mechanisms of exosome activity may arise from proteomic information. These exosome activities are directly associated with the very characteristics of medulloblastomas that make them so deadly–proliferation, migratory dissemination [4], and immune suppression [81], [82]. We have also identified a potentially novel tumor suppressor in medulloblastomas based on the presence of HNF4A in D283MED exosomes. While that and other aspects of our work clearly require further study, the data presented here indicate that tumor exosomes provide tumor cells with the capacities favoring the tumor in terms of growth, migration, and defense, as well as the possibility that exosomes may dispose of unwanted materials, which was what led to their original discovery [83]. The interactions between exosomes, tumor cells, the microenvironment, and the cadre of normal cells composing that environment, may ultimately reveal novel ways to target tumors such as medulloblastomas.

## Methods

### Ethics Statement for Human (patient/donor) Serum Collection and for Healthy Donor Peripheral Blood Mononuclear Cells (PBMCs) Acquisition

Blood was collected from pediatric patients at The Children’s Hospital undergoing neurosurgical resection of intracranial tumors. These were histologically confirmed to be medulloblastomas. Blood was collected in serum separator (tiger-top) tubes; tubes were centrifuged after clot formation, and sera were aliquoted and frozen at −80°C. PBMCs were harvested from spent blood collection cartridges obtained from healthy donors from the blood bank at the University of Colorado. All specimens were completely anonymized. The study was approved by the Colorado Combined Institutional Review Board (COMIRB), 95–100, and samples were collected following informed written consent.

### Cell Lines as Lysate and Exosomes Sources

All tissue culture products were from Gibco Invitrogen (Invitrogen/Life Technologies, Carlsbad, CA, USA). Exosomes were harvested from the medulloblastoma cell line D283MED (a gift from Dr Rajeev Vibhakar, University of Colorado Denver). This line is in the ATCC, and establishment of this line was described previously [36]. D283MED cells were converted to Knockout DMEM medium plus Serum Replacement, supplemented with 5 ng/ml bFGF and EGF, and 2 mM L-glutamine. These media do not contain fetal calf serum so there was no need to clear the media of bovine exosomes prior to use. For other experiments (eg, proliferation and migration), D283MED cells were grown in DMEM with 2 mM L-glutamate, 4.5 g/l glucose, 1 mM sodium pyruvate, and with 10% fetal bovine serum. UW228 medulloblastoma cells were also obtained from Dr Vibhakar and are described here [84]. DAOY medulloblastoma cells (HTB-186) and U87MG glioma cells (HTB-14) were obtained from the ATCC (Manassas, VA, USA). These cells were initially grown in DMEM/FBS but converted to Knockout medium as described above. Cells were grown at 1 million cells/ml in T-175 flasks with 25 ml Knockout medium. After 4 days in culture, 100 ml of media were collected and exosomes harvested as described. Final exosome concentrations ranged from 2.8–5.3 mg/ml (referenced to exosome protein content).

### Exosome Preparations

Based on previous use of filtration and concentrator devices for vesicle isolation [85], [86], we harvested exosomes from spent cell line media by filtration thru a 0.22 µm filter (Corning, Inc, Corning, NY, USA) followed by concentration in centrifugal devices with 100 kDa cutoff membranes (Millipore, Billerica, MA, USA). The concentrated material was centrifuged at 100,000 x g (4°C) in a Beckman 70.1 Ti rotor (Beckman Instruments, Fullerton, CA, USA) for 1 hour. The resulting pellet was resuspended in 8 ml phosphate buffered saline (PBS), and pelleted again at 100,000 x g, 1 hr as above. The final pellet was resuspended in a small volume of PBS and was quantified as described [29]. Exosome preparations were stored at 4°C until used.

### ExoQuick Precipitation of Serum Exosomes

We isolated exosomes from sera (or plasma) from patients with medulloblastomas or from healthy donors by using ExoQuick precipitation [87] (System Biosciences Inc, Mountain View, CA) following manufacturer’s instructions. Equal volumes of sera from patients or healthy donors were precipitated and extracted, and equal volumes of protein samples were loaded on SDS-PAGE gels for electrophoresis and Western blotting as described below.

### Exosome Characterizations

#### Dynamic light scattering (DLS)

D283 exosomes were aliquoted into 400 µl samples and transferred to a cuvette for size measurement using dynamic light scattering analysis. A particle size distribution was determined by a Nicomp 370 submicron particle sizer (Agilent Technologies Inc., Particle Sizing Systems Division, Santa Barbara, CA, USA). The Nicomp Zpw software adjusted the channel width for each sample based on the fluctuation rate of scattered light, and the final size distribution for each sample was calculated using the number-weight Gaussian setting within the software.

#### NanoSight

Size distributions of D283 exosome preparations and quantification of them were determined by measuring the rate of Brownian motion using a NanoSight LM10 system, which is equipped with a fast video capture and particle-tracking software (NanoSight Ltd, Wiltshire, UK).

#### Density gradient centrifugation

Exosomes were subjected to density gradient centrifugation for buoyant density determination on an OptiPrep (Axis-Shield; Greiner Bio One Inc., Monroe, NC, USA) gradient as descried previously [17]. Exosomes were prepared in 20 mM HEPES buffer, pH 7.4, and spun at 100,000 x g for 18 h in a Beckman SW-41 rotor. The gradient consisted of steps of 0–60% OptiPrep (in 20 mM HEPES buffer, pH 7.4). One-milliliter fractions were collected, fraction densities were determined, and other analyses (acetylcholinesterase activity, electron microscopy) were performed (described below).

#### Acetylcholinesterase (AChE) activity and transmission electron microscopy (TEM)

AChE activity of the OptiPrep fractions was performed as before [17]. Fractions were analyzed by electron microscopy after incubation on formar-coated grids, and negative staining with uranyl acetate, and observation with a Philips transmission electron microscope (Philips, Best, The Netherlands) operated at 80 kV as described [29]. For ExoQuick-precipitated serum exosomes, pellets were resuspended in100 µl dilute (0.1x) PBS and were further diluted 1∶100 in water before uranyl acetate staining. Those exosomes were examined and imaged with a Technai G2 equiped with a Gatan Ultrascan digital camera from FEI (Hillsboro, OR).

#### Free-solution isoelectric focusing

Free-solution isoelectric focusing was performed as described [17] using a Rotofor device (Bio-Rad Laboratories, Hercules, CA, USA). OptiPrep fractions containing exosomes were diluted to 30 ml in water that contained 0.25% (v/v) each of Fluka High-Resolution carrier ampholytes, pH ranges 3–10, 3–6, 4–6, 5–7 (Sigma-Aldrich, St Louis, MO, USA). The sample was loaded into the focusing chamber (0.1M H_3_PO_4_, anode; 0.1M NaOH, cathode). Isoelectric focusing was conducted at 15W constant power for 4 hr. Twenty fractions were harvested, and the pH of each fraction was measured. AChE assays were performed on each fraction.

#### Proteomic Analyses

D283MED exosomes were separated by SDS-PAGE as described [17], [29]; entire lanes of 1-D gels were cut into strips, and 1 cm segments of the gel lanes were cut as fractions for trypsin digests as described [88]. Proteins were processed for protein identification by tandem mass spectrometry at the Duke-UNC Michael Hooker Proteomics Center of the University of North Carolina, Chapel Hill, as described [88], [89] as we have done previously [17]. The MS and MS/MS spectra were used for protein identification using the non-redundant protein database NCBInr [90] and the human IPI protein database (version 3.71) consisting of 173490 entries; these were searched using the Mascot search engine, Version 2.2 (http://www.matrixscience.com/). All protein “hits” had MOWSE scores ≥35 (via Mascot) or ≥95 for MS/MS peptide ion fragment matches. There were at least 2 peptides per hit, and the protein had to be positively identified in 2 separate experiments. Protein subcellular localizations and functions were determined from literature searches and Ingenuity Systems software (Redwood City, CA, USA; http://www.ingenuity.com/index.html). Pathway analyses and network constructions were assembled using the Ingenuity software.

#### Western blots and fluorescence activated cell sorting (FACS)

Western blots of D283MED exosomes and FACS (with the exosomes adhered to latex beads) were performed as described [17], [29] for HSPs 90, 70, and 27 (including HSC70), protein disulfide isomerase, alpha-1 antitrypsin, GAPDH; for bead FACS, the fluorescently-labeled HSP90 monoclonal antibody SPA-830 (Nventa Biopharmaceuticals, San Diego, CA, USA) was used. Other antibodies used in these studies: against HSP60, SPA-807 (Nventa Biopharmaceuticals); against hemopexin, ab90947 (Abcam, Cambridge, MA, USA); against HNF4A, PP-K9218-00 (Perseus Proteomics, Tokyo, Japan); against Her2/ErbB2, #2242 (Cell Signaling Technologies, Danvers MA, USA); against glycoprotein non-metastatic B (GPNMB), ab98856 (Abcam); against CD9 and CD63, EXOAB-CD9A-1 and EXOAB-CD63A-1, respectively (Systems Biosciences Inc). D283MED, DAOY, UW228, and U87MG cells were lysed as described [17], where we pointed out that brain tumor exosomes are quite resistant to most detergent-based lysis buffers. Briefly, exosome pellets were diluted in 100 µl PBS and then were supplemented with 100 µl phenol and vortexed. The mixture was heated to 70°C for 10 min in a safety hood, and transferred to ice for 5 min, followed by centrifugation (5000 *g*, 10 min). The aqueous phase was discarded, and these steps were repeated by adding 100 µl diH2O. After spinning, the aqueous phase was discarded, 200 µl of acetone was added, vortexed, and spun as before. The supernatant was discarded, the previous step was repeated, and the resulting pellet was air-dried. CD9 positive control cell lysates A20 was from Abcam (ab-7180). Lysates are loaded at 20 µg per lane; exosomes are loaded at maximum volume (30 µl, of which half is protein sample), so direct comparisons of protein load or enrichment are not necessarily applicable.

### Proliferation/Metabolic Assays

#### MTS assay

D283MED cells were plated in 50 µl DMEM +10% FBS at a concentration of 10,000 cells/well in a 96 well plate. D283MED exosomes were added at concentrations of 0, 5, 50, and 100 µg/ml (in 50 µl DMEM+FBS), and cells were allowed to proliferate for 24, 48, 72, and 92 hrs. At the end of the growth period, 20 µl Cell Titer 96 AQueous One Solution [3-(4,5-dimethylthiazol-2-yl)-5-(3-carboxymethoxyphenyl)-2-(4-sulfophenyl)-2H-tetrazolium, MTS, in the presence of phenazine ethosulfate (PES)] (Promega, Madison, WI, USA) was added for 1 hr, and absorbance was read at 490 nm in a Hidek Chameleon Plate Reader (Bioscan, Inc, Washington, DC, USA). Increased reduction to formazan product was plotted as percent of control cells.

#### ATP assay

D283MED cells were plated in a 96 well plate at 5000 or 10,000 cells per well (50 µl) in DMEM/FBS as above. Exosomes were added at concentrations of 0, 5, 25, 50, 100, and 500 µg/ml (in 50 µl DMEM/FBS), and cells were allowed to proliferate for 24 and 48 hrs. At the end of the growth period, 100 µl Cell Titer Glo reagent (Promega) was added and incubated for 1.5 hrs. In this assay luciferase catalyzes the oxygenation of luciferin in the presence of ATP (proportional to cell number), with light emission. Liquid was transferred to a luminometer plate, and luminescence was quantified in “glow” mode (integration time of 1 sec, direct photon counting) in a Chameleon Plate Reader (Bioscan). Three individual experiments for proliferation assays were performed and the data combined, displayed as average values +/− standard deviation.

#### Migration assay

D283MED, UW228, and DAOY cells grown in DMEM/FBS were cultured overnight in serum-containing, or serum-free DMEM, and plated at 100,000 cells per well in the upper well of a Boyden chamber (CytoSelect Cell Migration Assay, Cell Biolabs, Inc, San Diego, CA, USA). Cells were separated from the lower chamber by a polycarbonate filter (8 µm pore size), and the lower chamber medium consisted of DMEM only, DMEM +10% FBS as a positive control attractant, or DMEM +50, 100, or 500 µg/ml D283MED, or UW228, or DAOY exosomes. Cells were allowed to migrate for 48 hrs, after which the remaining cells were removed from the upper chamber and the insert was stained and washed. Migratory cells were counted on the “bottom” side of the insert in 3 high-power fields (40X, duplicates of each condition); average numbers of cells per field per condition (+/− standard deviation) were plotted. Data from 2 or 3 experiments were combined.

#### Interferon-γ Release Assay

Interferon-γ release from peripheral blood mononuclear cells (PBMCs) was evaluated by ELISA (Human IFNγ ELISA kit, Pierce Thermo Scientific, Rockford, IL, USA). Healthy donor PBMCs were acquired from spent blood cassettes obtained thru a local blood bank. PBMCs were harvested by standard Ficoll separation and were incubated in DMEM/FBS in triplicate at 100,000 cells/well in 48 well plates. Phytohemagglutinin (PHA, Sigma-Aldrich) was added as a stimulant at 5 µg/ml, and D283MED exosomes were added at concentrations of 0, 5, 25, 50, 100, and 500 µg/ml for 48 hrs. Supernatants were collected and evaluated for IFN-γ via ELISA; IFN-γ quantities were determined by comparison with a standard curve. Assays were performed in quadruplicate for two separate donors; data were combined and outputs averaged (+/− standard deviation).

#### Clonogenicity assays and treatment with MEDICA 16 and exosomes

D283MED suspension cells and UW228, DAOY adherent cells were grown in DMEM +10% FBS to exponential growth. For clonogenic assays, we used the Cyto-select 96-well Tumor Sensitivity Assay (Cell Biolabs catalog # CBA-150), according to the recommended guidelines. Briefly, a soft agar base layer was prepared consisting of equal parts 1.2% agar solution and 2X DMEM +20% FBS. Fifty µl of this base layer was allocated into a standard 96-well, flat-bottomed plate, which was then stored at 4°C for 30 min to solidify the agar. A cell agar layer was then prepared of equal parts 1.2% agar solution, DMEM +20% FBS, and cell suspensions containing 4.0×10^5^ cells/ml. Seventy-five µl of this cell agar layer was then immediately applied to the base layer in each well. The plate was then stored at 4°C/15 min to allow solidification of the cell agar layer. The following treatments were then prepared to a total volume of 100 µL using DMEM as the diluent, for plating of 8 replicates per condition: D283MED exosomes at concentrations of 25 or 100 µg/ml, +/− MEDICA 16 (Cayman Chemicals, catalog # 90290) at a concentrations of 125 µM. Plates were incubated at 37°C and 5% CO_2_ for 8 days, with cell colony formation monitored by microscopy. For standard clonogenicity assays, we also used UW228 and DAOY cells, and 50–500 µg/ml cognate exosomes were used as growth stimulants. For quantitative analysis, Cyto-select Invitrogen Sensitivity Assay agar solubilization solution was added to each well and gently mixed. The plate was incubated for one hr/37°C, followed by repeated pipette mixing of each well. Kit lysis buffer was then applied to each well with repeated pipetting. The plate was incubated at room temperature for 15 min; 10 µl from each well was transferred to a new 96 well, flat-bottomed plate. Kit Cyquant working solution was added to each well, and the plate was incubated at room temperature for 10 min, and fluorescence readings recorded using a Chameleon Plate Reader set at the 520 nm filter setting. Experiments were performed twice and data were combined, averaged, and statistically analyzed.

### Statistical Analyses

Where applicable, Student *t* test was used for comparisons to determine statistical significance; in other cases, data were analyzed by analysis of variance (ANOVA) followed by Tukey’s post hoc multiple comparison tests (SPSS 20, (http://www-01.ibm.com/software/analytics/spss/?pgel=ibmhzn&cm_re=masthead-_-products-_-sw-sps), where p<0.05 was chosen as significant unless otherwise stated. Error bars in all cases depict standard deviation. Statistics used for IPA can be found at the website http://www.ingenuity.com/index.html.

## Supporting Information

Figure S1 **Extended list of Top Network Functions/Biofunctions: “Diseases and Disorders” from IPA Core Analysis.** This list encompasses the top 72 categories with scores above the threshold for significance. “Threshold” indicates the minimum significance level (scored as –log [p value] from Fisher’s exact test, set here at 1.25).
(TIF)

Figure S2 **Extended list of the Top Network Functions “Top Canonical Pathways” from IPA Core Analysis.** This list encompasses the top 28 categories with scores above the threshold for significance. “Threshold” indicates the minimum significance level (scored as –log [p value] from Fisher’s exact test, set here at 1.25). “Ratio” indicates the number of molecules from the data set that map to the pathway listed divided by the total number of molecules that map to the canonical pathway from within the IPA database.
(TIF)

Figure S3 **Top Network Functions “Top Toxicology Functions and Lists” from IPA Core Analysis.** (**A**) shows the top 12 significantly-scoring toxicology functions derived from IPA analyses; (**B**) shows the top 5 toxicology lists. The statistically significant threshold is defined as in **Figures S1** and **S2**.
(TIF)

Table S1 **Results of D283MED exosome proteomics.** Table lists proteins identified by mass spectrometry or Western blot analyses. Gene/protein IDs, symbols, and names are presented, along with peptide count, source of identification (MS or Western blot), proteins’ predicted subcellular localizations and putative functions, and presence in the ExoCarta database. Details are at the bottom of the Table.
(DOC)
