# Supplementary figures and images for: Medulloblastoma Exosome Proteomics Yield Functional Roles for Extracellular Vesicles
Source: PLoS One. 2012 Jul 27;7(7):e42064. doi: 10.1371/journal.pone.0042064 (PMC3407172; doi:10.1371/journal.pone.0042064)

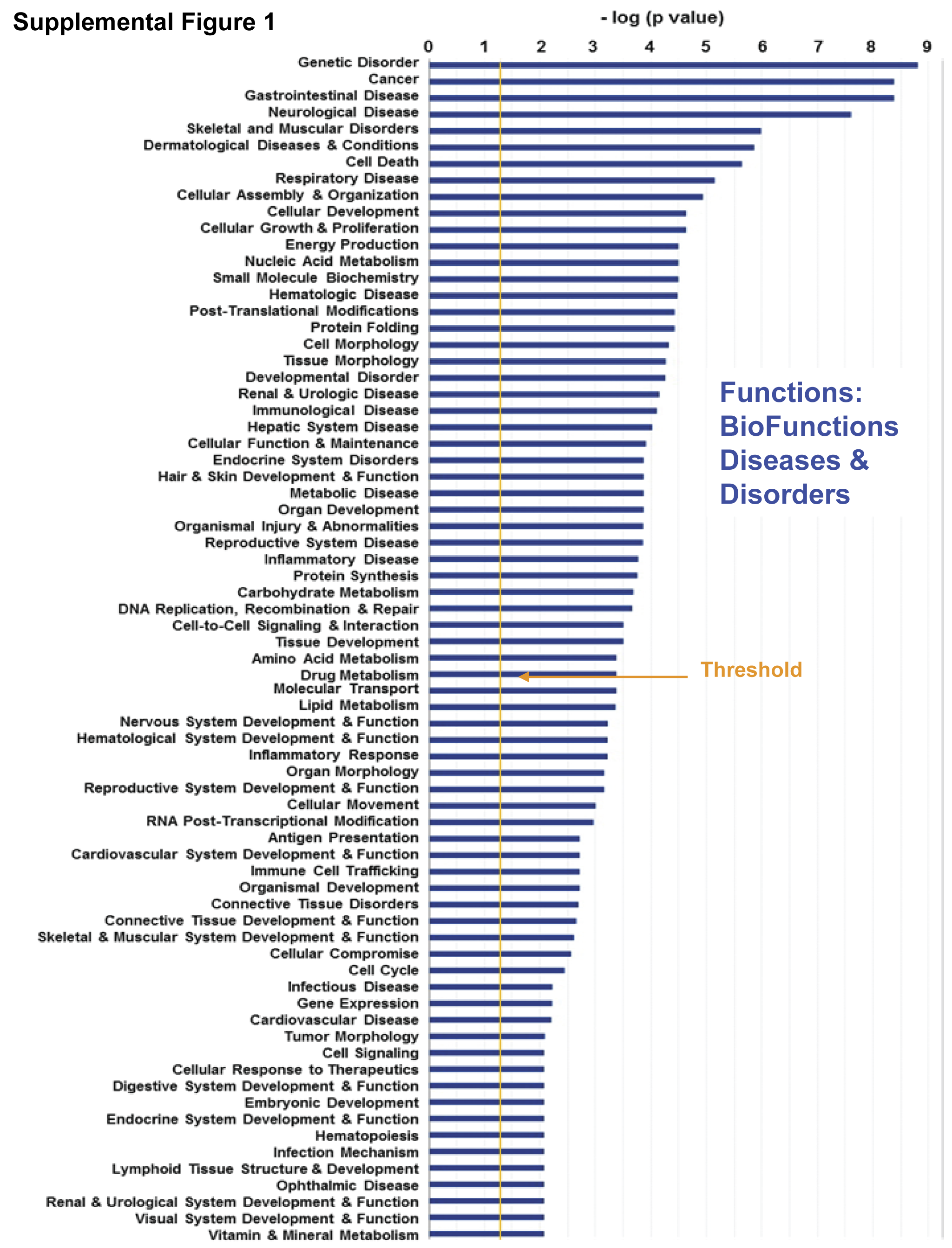

Supplement: Figure S1 — Extended list of Top Network Functions/Biofunctions: “Diseases and Disorders” from IPA Core Analysis. This list encompasses the top 72 categories with scores above the threshold for significance. “Threshold” indicates the minimum significance level (scored as –log [p value] from Fisher’s exact test, set here at 1.25). (TIF) [file pone.0042064.s001.tif]

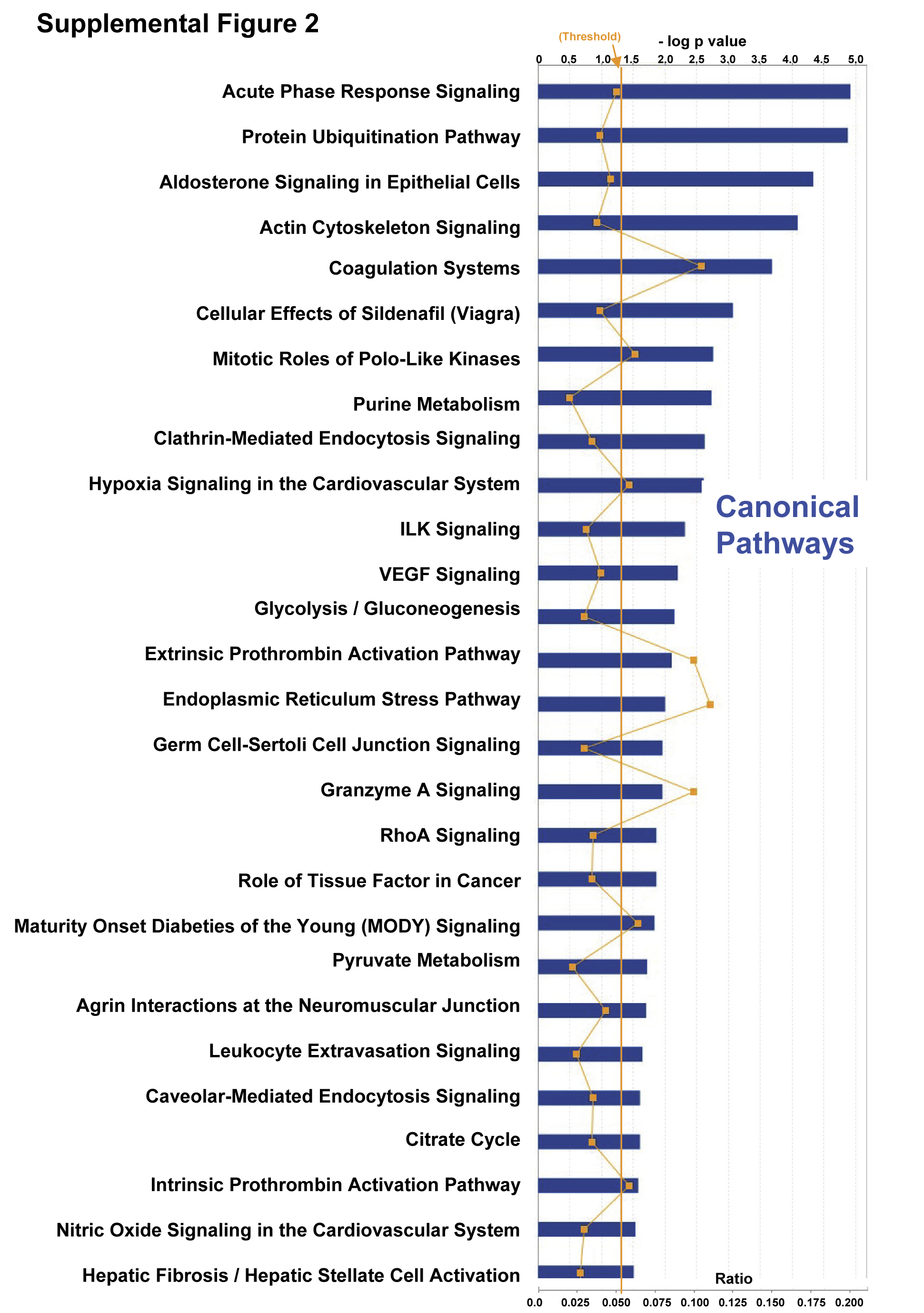

Supplement: Figure S2 — Extended list of the Top Network Functions “Top Canonical Pathways” from IPA Core Analysis. This list encompasses the top 28 categories with scores above the threshold for significance. “Threshold” indicates the minimum significance level (scored as –log [p value] from Fisher’s exact test, set here at 1.25). “Ratio” indicates the number of molecules from the data set that map to the pathway listed divided by the total number of molecules that map to the canonical pathway from within the IPA database. (TIF) [file pone.0042064.s002.tif]

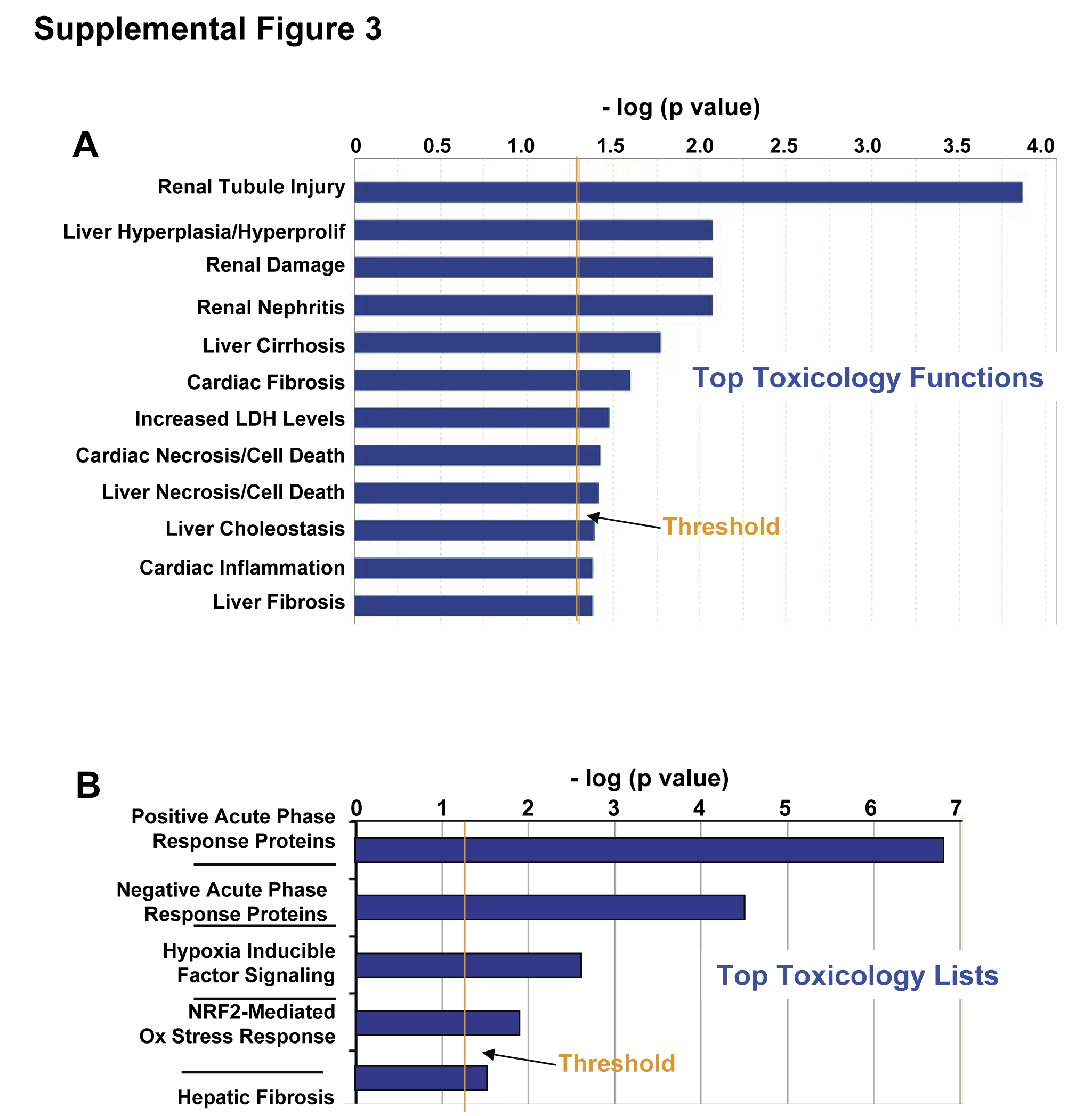

Supplement: Figure S3 — Top Network Functions “Top Toxicology Functions and Lists” from IPA Core Analysis. (A) shows the top 12 significantly-scoring toxicology functions derived from IPA analyses; (B) shows the top 5 toxicology lists. The statistically significant threshold is defined as in Figures S1 and S2. (TIF) [file pone.0042064.s003.tif]
