# Supplementary material for: Medulloblastoma Exosome Proteomics Yield Functional Roles for Extracellular Vesicles
Source: PLoS One. 2012 Jul 27;7(7):e42064. doi: 10.1371/journal.pone.0042064 (PMC3407172; doi:10.1371/journal.pone.0042064)
Supplement: Table S1 — Results of D283MED exosome proteomics. Table lists proteins identified by mass spectrometry or Western blot analyses. Gene/protein IDs, symbols, and names are presented, along with peptide count, source of identification (MS or Western blot), proteins’ predicted subcellular localizations and putative functions, and presence in the ExoCarta database. Details are at the bottom of the Table. (DOC) [file pone.0042064.s004.doc]

**Table S1: Results of Combined Mass Spec and Immunotblot Proteomic Analyses from D283MED Medulloblastoma Exosomes**

| **ID** | **Symbol** | **Entrez Gene Name** | **PepCt** | **MS/WB** | **Loc** | **Type** | **ExoCarta?** |
| --- | --- | --- | --- | --- | --- | --- | --- |
| P01023 | A2M | Alpha-2-Macroglobulin | 4 | MS | ECS | PI | Yes P |
| P78363 | ABCA4 | ATP-binding cassette, sub-family A  (ABC1), member 4 | 9 | MS | PM | Transport | No |
| Q9Y305 | ACOT9 | Acyl-CoA thioesterase 9 | 2 | MS | Mito | Enz | No |
| P60709 | ACTB | Actin, beta | 11 | MS/WB* | Cyto | Struct | Yes P |
| P63261 | ACTG1 | Actin, gamma 1 | 9 | MS | Cyto | Struct | Yes P |
| P35609 | ACTN2 | Actinin, alpha 2 | 26 | MS | Cyto | Txn Regl | Yes P |
| Q76LX8 | ADAMTS13 | A disintegrin and metalloproteinase with thrombospondin motifs 13 | 2 | MS | ECS | Protease | No |
| P02768 | ALB | Albumin (human) | 8 | MS | ECS | Transport | Yes P |
| P04075 | ALDOA | Aldolase A, fructose-bisphosphate | 20 | MS | Cyto | Enz | Yes P |
| P07355 | ANXA2 | Annexin A2 | 4 | MS | PM | Scaf | Yes P |
| P27695 | APEX1 | APEX nuclease  (multifunctional DNA repair enzyme) 1 | 4 | MS | Nuc | Enz | Yes P |
| [P02647](http://www.uniprot.org/uniprot/P02647) | APOA1 | Apolipoprotein A-I | 3 | MS | ECS | Transport | Yes P |
| P06727 | APOA4 | Apolipoprotein A-IV | 2 | MS | ECS | Transport | Yes P |
| Q9NWB6 | ARGLU1 | Arginine and glutamate rich 1 | 2 | MS | Nuc | Txn Regl | No |
| P00966 | ASS1 | Argininosuccinate synthetase 1 | 26 | MS | Cyto | Enz | Yes P |
| P06576 | ATP5B | ATP synthase, H+ transporting,  mitochondrial F1 complex, beta polypeptide | 11 | MS | Mito | Transport | Yes P |
| Q99728 | BARD1 | BRCA1 associated RING domain 1 | 10 | MS | Nuc | Txn Regl | No |
| Q9NR09 | BIRC6 | Baculoviral IAP repeat-containing protein 6 | 2 | MS | Cyto | Enz | Yes R |
| P51451 | BLK | B lymphoid tyrosine kinase | 2 | MS | Cyto | Kinase | No |
| P01024 | C3 | Complement C3 | 4 | MS | ECS | Protease | Yes P |
| P62158 | CALM1/2/3 | Calmodulin | 4 | MS | Cyto | Scaf | Yes P |
| Q9UL16 | CCDC19 | Coiled-coil domain containing protein 19 | 13 | MS | Mito | Other | No |
| Q8IWP9 | CCDCC28A | Coiled-coil domain-containing protein 28A | 2 | MS | Unk | Other | No |
| P21926 | CD9 | CD9 molecule, Tetraspanin-29 | -- | WB | PM | Scaf | Yes P |
| Q02224 | CENPE | Centromere protein E, 312kDa | 2 | MS | Nuc | Motor | No |
| P41208 | CETN2 | Centrin, EF-hand protein, 2 | 2 | MS | Nuc | Enz | No |
| Q14993 | COL19A1 | Collagen, type XIX, alpha 1 | 19 | MS | ECS | Struct | No |
| P19784 | CSNK2A2 | Casein kinase 2, alpha prime polypeptide | 2 | MS | Cyto | Kinase | Yes P |
| Q13618 | CUL3 | Cullin 3 | 8 | MS | Nuc | Enz | Yes P |
| Q93034 | CUL5 | Cullin 5 | 18 | MS | Nuc | Transport | Yes P |
| Q08211 | DHX9 | DEAH (Asp-Glu-Ala-His) box polypeptide 9 | 15 | MS | Nuc | Enz | Yes P |
| Q9NX36 | DNAJC28 | DnaJ (Hsp40) homolog, subfamily C, member 28 | 26 | MS | Cyto | Chap | No |
| Q99615 | DNAJC7 | DnaJ (Hsp40) homolog, subfamily C, member 7 | 6 | MS | Cyto | Chap | Yes P |
| Q96N67 | DOCK7 | Dedicator of cytokinesis protein 7 | 2 | MS | PM | Enz | No |
| Q14195 | DPYSL3 | Dihydropyrimidinase-like 3 | 2 | MS | Cyto | Enz | Yes P |
| P15924 | DSP | Desmoplakin | 12 | MS | PM | Scaf | Yes P |
| Q14222 | EEF1A1 | Eukaryotic translation elongation factor 1 alpha 1 | 3 | MS | Cyto | Tznl Regl | Yes P |
| P13639 | EEF2 | Eukaryotic translation elongation factor 2 | 9 | MS | Cyto | Tznl Regl | Yes P |
| P05198 | EIF2S1 | Eukaryotic translation initiation factor 2,  subunit 1 alpha, 35kDa | 10 | MS | Cyto | Tznl Regl | Yes P |
| Q14152 | EIF3A | Eukaryotic translation initiation factor 3,  subunit A | 23 | MS | Cyto | Tznl Regl | Yes P |
| P06733 | ENO1 | Enolase 1, (alpha) | 22 | MS | Cyto | Enz | Yes P |
| P04626 | ERBB2 | Receptor tyrosine protein kinase erbB-2  (HER2/NEU, CD340) | -- | WB | PM | Kinase | Yes P |
| Q6NUR7 | EZR | Ezrin | 6 | MS/WB | PM | Scaf | Yes P |
| P02671 | FGA | Fibrinogen alpha chain | 2 | MS | ESS | Struct | Yes P |
| P02675 | FGB | Fibrinogen beta chain | 4 | MS | ESS | Struct | Yes P |
| P09038 | FGF2 | Heparin-binding growth factor 2/basic fibroblast  growth factor | 4 | MS | ESS | Horm/GF | No |
| Q5SYB0 | FRMPD1 | FERM and PDZ domain-containing protein 1 | 5 | MS | Cyto | Struct | No |
| Q96AE4 | FUBP1 | Far upstream element (FUSE) binding protein 1 | 2 | MS | Nuc | Txn Regl | No |
| P04406 | GAPDH | Glyceraldehyde-3-phosphate dehydrogenase | 13 | MS/WB | Cyto | Enz | Yes P |
| P14136 | GFAP | Glial fibrillary acidic protein | 18 | MS | Cyto | Struct | No |
| P01286 | GHRH | Growth hormone releasing hormone | 7 | MS | ESS | Horm/GF | No |
| Q14956 | GPNMB | Glycoprotein (transmembrane) nmb | 6 | MS/WB | PM | Enz | Yes P |
| Q5VW38 | GPR107 | G protein-coupled receptor 107 | 2 | MS | PM | Enz | Yes R |
| P06396 | GSN | Gelsolin (AGEL) | 17 | MS | Cyto | Struct | Yes P |
| Q9BZE4 | GTPBP4 | GTP binding protein 4 | 2 | MS | Nuc | Enz | Yes P |
| P0C0S5 | H2AFZ | Histone H2A.Z | 2 | MS | Nuc | NA Bind | Yes PR |
| P69905 | HBA1 HBA2 | Hemoglobin subunit alpha | 4 | MS | ECS | Transport | Yes P |
| P68871 | HBB | Hemoglobin subunit beta | 2 | MS | ECS | Transport | Yes P |
| P0C0S8 | HIST1H2AG,  I, K, L, M | Histone H2A type 1 AG, AI, AK, AL, AM | 2 | MS | Nuc | NA Bind | Yes P |
| P06899 | HIST1H2BJ | Histone H2B type 1-J | 5 | MS | Nuc | NA Bind | Yes P |
| P68431 | HIST1H3A | Histone H3.1 A-J | 3 | MS | Nuc | NA Bind | Yes P |
| P41235 | HNF4A | Hepatocyte nuclear factor 4 alpha | -- | WB | Nuc | Txn Regl | No |
| P52272 | HNRNPM | Heterogeneous nuclear ribonucleoprotein M | 18 | MS | Nuc | NA Bind | No |
| Q00839 | HNRNPU | Heterogeneous nuclear ribonucleoprotein U  (scaffold attachment factor A) | 18 | MS | Nuc | NA Bind | Yes R |
| O14979 | HNRPDL | Heterogeneous nuclear ribonucleoprotein D-like | 2 | MS | Nuc | NA Bind | No |
| P02790 | HPX | Hemopexin (Beta 1B-glycoprotein) | 18 | MS | ECS | Transport | Yes P |
| O75322 | HSP90AA1 | Heat shock protein 90kDa alpha (cytosolic),  class A member 1 | 21 | MS/WB | Cyto | Chap | Yes PR |
| P08238 | HSP90AB1 | Heat shock protein 90kDa alpha (cytosolic),  class B member 1 | 2 | MS/WB | Cyto | Chap | Yes P |
| P08107 | HSPA1B | Heat shock 70kDa protein 1B (testis specific) | 8 | MS | Cyto | Chap | Yes P |
| P11021 | HSPA5 | Heat shock 70kDa protein 5  (glucose-regulated protein, 78kDa) | 15 | MS | ER | Chap | Yes P |
| P11142 | HSPA8 | Heat shock 70kDa protein 8 (cognate 71 kDa) | 2 | MS/WB | Cyto | Chap | Yes P |
| P04792 | HSPB1 | Heat shock 27kDa protein 1 | 10 | MS/WB | Cyto | Chap | Yes P |
| P10809 | HSPD1 | Heat shock protein 60 kDa, chaperonin 60 | 5 | MS/WB | Mito | Chap | Yes P |
| O75874 | IDH1 | Isocitrate dehydrogenase 1 (NADP+), soluble | 8 | MS | Cyto | Enz | Yes P |
| P01591 | IGJ | Immunoglobulin J chain | 9 | MS | ECS | Other | Yes P |
| P01621 |  | Ig kappa chain V-III region NG9 | 20 | MS | ECS | Other | ------ |
| P01700 |  | Ig lambda chain V-I region HA | 4 | MS | ECS | Other | ------ |
| O43526 | KCNQ2 | Potassium voltage-gated channel subfamily KQT member 2 | 2 | MS | PM | Transport | No |
| Q07666 | KHDRBS1 | KH domain containing, RNA binding,  signal transduction associated 1 | 6 | MS | Nuc | Txn Regl | No |
| Q92945 | KHSRP | KH-type splicing regulatory protein | 21 | MS | Nuc | Enz | No |
| Q96L93 | KIF16B | Kinesin family member 16B | 4 | MS | Cyto | Motor | No |
| P04264 | KRT1 | Keratin type II cytoskeletal 1 | 17 | MS | Cyto | Struct | Yes P |
| P35908 | KRT2 | Keratin, type II cytoskeletal 2 epidermal | 9 | MS | Cyto | Struct | Yes P |
| P05787 | KRT8 | Keratin type II cytoskeletal 8 | 13 | MS | Cyto | Struct | Yes P |
| P35527 | KRT9 | Keratin, type I cytoskeletal 9 | 9 | MS | Cyto | Struct | Yes P |
| P13645 | KRT10 | Keratin, type I cytoskeletal 10 | 14 | MS | Cyto | Struct | Yes P |
| P02533 | KRT14 | Keratin, type I cytoskeletal 14 | 4 | MS | Cyto | Struct | Yes P |
| P32004 | L1CAM | Neural cell adhesion molecule L1  (NCAM-L1, CD171) | -- | WB* | PM | Struct | Yes PR |
| Q8NES3 | LFNG | LFNG O-fucosylpeptide  3-beta-N-acetylglucosaminyltransferase | 3 | MS | Cyto | Enz | Yes P |
| Q8N2S1 | LTBP4 | Latent-transforming growth factor β-binding  protein 4 | 2 | MS | ECS | Horm/GF | No |
| Q9UJA3 | MCM8 | Minichromosome maintenance complex  component 8 | 2 | MS | Nuc | Enz | No |
| Q75MT9 | MDH2 | Malate dehydrogenase 2, NAD (mitochondrial) | 9 | MS | Mito | Enz | Yes P |
| Q13201 | MMRN1 | Multimerin 1 | 2 | MS | ECS | Struct | Yes P |
| Q9BRJ2 | MRPL45 | Mitochondrial ribosomal protein L45 | 10 | MS | Mito | Tznl Regl | No |
| P35580 | MYH10 | Myosin, heavy chain 10, non-muscle | 29 | MS | Cyto | Motor | Yes P |
| P35579 | MYH9 | Myosin, heavy chain 9, non-muscle | 26 | MS | Cyto | Motor | Yes P |
| O94832 | MYO1D | Myosin ID | 22 | MS | PM | Motor | Yes P |
| Q9P032 | NDUFAF4 | NADH dehydrogenase [ubiquinone] 1 alpha subcomplex assembly factor 4 | 9 | MS | Mito | Enz | No |
| P20929 | NEB | Nebulin | 27 | MS | Cyto | Struct | Yes P |
| O76041 | NEBL | Nebulette | 11 | MS | PM | Struct | Yes P |
| Q14511 | NEDD9 | Enhancer of filamentation 1 | 2 | MS | Nuc | Scaf | No |
| Q9NQ35 | NRIP3 | Nuclear receptor interacting protein 3 | 2 | MS | Nuc | Protease | No |
| P07237 | P4HB | Prolyl 4-hydroxylase, beta polypeptide | 10 | MS/WB | ER | Chap | Yes P |
| Q9NV79 | PCMTD2 | Protein-L-isoaspartate O-methyltransferase  domain-containing protein 2 | 2 | MS | Cyto | Enz | No |
| O95613 | PCNT | Pericentrin | 38 | MS | Cyto | Other | No |
| P16499 | PDE6A | Phosphodiesterase 6A, cGMP-specific, rod, α | 2 | MS | PM | Enz | No |
| O43175 | PHGDH | Phosphoglycerate dehydrogenase | 7 | MS | Cyto | Enz | Yes P |
| Q00169 | PITPNA | Phosphatidylinositol transfer protein, α | 2 | MS | Cyto | Transport | No |
| P14618 | PKM2 | Pyruvate kinase, muscle | 10 | MS | Cyto | Kinase | Yes P |
| Q9NYY3 | PLK2 | Polo-like kinase 2 (Drosophila) | 12 | MS | Nuc | Kinase | No |
| P20664 | PRIM1 | Primase, DNA, polypeptide 1 (49kDa) | 8 | MS | Nuc | Enz | No |
| P49720 | PSMB3 | Proteasome (prosome, macropain)  subunit, beta type, 3 | 9 | MS | Cyto | Protease | Yes PR |
| P62195 | PSMC5 | Proteasome (prosome, macropain)  26S subunit, ATPase, 5 | 24 | MS | Nuc | Txn Regl | Yes P |
| P11216 | PYGB | Phosphorylase, glycogen; brain | 14 | MS | Cyto | Enz | Yes P |
| Q9H0U4 | RAB1B | RAB1B, member RAS oncogene family | 11 | MS | Cyto | Enz | Yes P |
| P35241 | RDX | Radixin | 6 | MS | Cyto | Other | Yes P |
| P35251 | RFC1 | Replication factor C (activator 1) 1, 145kDa | 22 | MS | Nuc | Txn Regl | Yes P |
| Q8TAA1 | RNASE11 | Ribonuclease, RNase A family, 11 (non-active) | 3 | MS | ECS | Other | Yes P |
| Q9Y4L5 | RNF115 | Ring finger protein 115 | 5 | MS | Cyto | Other | No |
| P05387 | RPLP2 | Ribosomal protein, large, P2 | 4 | MS | Cyto | Other | Yes PR |
| P23396 | RPS3 | Ribosomal protein S3 | 2 | MS | Cyto | Other | Yes P |
| O95171 | SCEL | Sciellin | 3 | MS | Cyto | Other | Yes P |
| P01009-2 | SERPINA1 | Serpin peptidase inhibitor, clade A  (alpha-1 antiproteinase, antitrypsin), member 1 | 4 | MS/WB | ECS | Other | Yes P |
| P05543 | SERPINA7 | Thyroxine-binding globulin, serpin A7 | 2 | MS | ECS | PI | Yes P |
| Q01105 | SET | SET nuclear oncogene | 2 | MS | Nuc | Ph’tase | Yes R |
| P48962 | SLC25A4 | Solute carrier family 25  (mitochondrial carrier;  adenine nucleotide translocator), member 4 | 4 | MS | Mitoc | Transport | Yes P |
| P51881 | SLC25A5 | Solute carrier family 25  (mitochondrial carrier;  adenine nucleotide translocator), member 5 | 6 | MS | Mito | Transport | Yes PR |
| P28370 | SMARCA1 | SWI/SNF related, matrix associated,  actin dependent regulator of chromatin,  subfamily a, member 1 | 23 | MS | Nuc | Txn Regl | No |
| Q14683 | SMC1A | Structural maintenance of chromosomes 1A | 22 | MS | Nuc | Transport | Yes P |
| Q9BQB4 | SOST | Sclerostin | 9 | MS | ECS | Other | Yes P |
| Q01082 | SPTBN1 | Spectrin, beta, non-erythrocytic 1 | 40 | MS | PM | Other | Yes P |
| Q16385 | SSX2 | Synovial sarcoma, X breakpoint 2 (A&B)  Cancer/testis antigen 5.2, HOM-MEL-40 | 2 | MS | Nuc | Txn Regl | No |
| Q9Y6Q2 | STON1 | Stonin 1 | 11 | MS | PM | Other | No |
| O00186 | STXBP3 | Syntaxin binding protein 3 | 3 | MS | PM | Transport | Yes P |
| Q9H5I1 | SUV39H2 | Histone-lysine N-methyltransferase SUV39H2 | 2 | MS | Nuc | Txn Regl | No |
| O60506 | SYNCRIP | Synaptotagmin binding,  cytoplasmic RNA interacting protein | 4 | MS | Nuc | NA Bind | No |
| Q9H7E2 | TDRD3 | Tudor domain containing 3 | 2 | MS | Nuc | Txn Regl | No |
| Q1HBA5 | TF | Transferrin | 19 | MS | ECS | Transport | Yes P |
| Q9NVH6 | TMLHE | Trimethyllysine hydroxylase, epsilon | 9 | MS | Mito | Enz | No |
| P42167 | TMPO | Thymopoietin | 3 | MS | Nuc | Scaf | No |
| P59282 | TPPP2 | Tubulin polymerization-promoting protein  family member 2 | 8 | MS | Cyto | Enz | No |
| Q969E3 | UCN3 | Urocortin 3 (stresscopin) | 3 | MS | ECS | Horm/GF | No |
| P63104 | YWHAZ | Tyrosine 3-monooxygenase/tryptophan  5-monooxygenase activation protein,  zeta polypeptide (14-3-3 zeta) | 2 | MS | Cyto | Enz | Yes PR |
| P17030 | ZNF25 | Zinc finger protein 25 | 12 | MS | Nuc | Txn Regl | No |
| Q9H4T2 | ZSCAN16 | Zinc finger and SCAN domain containing 16 | 2 | MS | Nuc | Txn Regl | No |

“ID”/Accession numbers are from the UniProt Knowledge Base (Swiss-Prot/TrEMBL, <http://us.expasy.org/sprot/>)

“Symbol” refers to the Entrez gene ID

“Entrez gene name” is a protein descriptor

“PepCt” peptide count from mass spectrometry

“MS/WB” displays whether the protein was identified by mass spectrometry (MS) and/or by Western blotting (WB). Note, * = protein was identified by Western blot in reference 17

“Loc” = location, refers to the generally accepted subcellular localization of the protein.

“Type” categorizes the proteins’ functions/activities

Abbreviations used: PI = protease inhibitor; Enz = enzyme; Struct = structural; Txn Regl = transcriptional regulator; Scaf = scaffold; Tznl Regl = translational regulator; Chap = chaperone; NA Bind = nucleic acid (DNA/RNA) binding; Horm/GF = hormone/growth factor; Ph’tase = phosphatase

“ExoCarta?” indicates if the protein is found in that database (<http://www.exocarta.org/>) or not (as of January 2012); “R” indicates database entry as messenger RNA, “P” indicates protein.

All protein “hits” had MOWSE scores >35 (via Mascot) or >95 for MS/MS peptide ion fragment matches.
